# Self-boosting targeted anticancer therapy *via* cancer cell self-reprogramming with GGT-targeting oxidative stress nanoamplifiers

**DOI:** 10.7150/thno.120954

**Published:** 2026-01-01

**Authors:** Sujin Kim, Suyeon Lee, Manseok Yang, Seungwon Jung, Nanhee Song, Nuri Kim, Hanui Jo, Seunga Lee, Chaihong Nah, Seong-Cheol Park, Dongwon Lee

**Affiliations:** 1 Department of Bionanotechnology and Bioconvergence Engineering, Jeonbuk National University, Jeonju, Jeonbuk 54896, Republic of Korea.; 2 Department of Chemistry, Lehigh University, Bethlehem, PA 18015, USA.; 3 Department of Polymer Engineering, Sunchon National University, Sunchon, Jeonnam 57922, Republic of Korea.; 4 Department of PolymerNano Science and Technology, Jeonbuk National University, Jeonju, Jeonbuk 54896, Republic of Korea.

**Keywords:** reactive oxygen species, cancer, cancer cell reprogramming, gamma glutamyl transferase, targeted delivery

## Abstract

**Rationale:** *Gamma*-glutamyl transferase (GGT) is overexpressed on cancer cell membranes and has been widely used as a promising target for receptor-mediated therapy. However, its heterogeneous expression limits targeting efficacy. Based on the notation that reactive oxygen species (ROS) upregulate GGT and induce oxidative stress-mediated cancer cell death, we hypothesized that GGT-targeted ROS generation could simultaneously induce cell death and also reprogram tumors to achieve self-boosting targeted therapy.

**Methods:** We developed GLOXmp, a glutamic acid (Glu)-coated oxidative stress nanoamplifier, in which glutathione (GSH)-depleting B2C was loaded in ROS-generating amphiphilic polyCA. GLOXmp was designed to induce oxidative stress, modulate GGT expression, subsequently enhancing tumor targeting both in vitro and in vivo using xenograft mouse models.

**Results:** GLOXmp internalized GGT-overexpressing cancer cells and concurrently generated ROS and depleted intracellular GSH, leading to mitochondrial damage and potent cancer cell death. Importantly, GLOXmp reprogrammed tumor cells to upregulate GGT, leading to the enhancement of receptor-mediated uptake of subsequent doses. In tumor xenograft model, repeated administration of GLOXmp significantly elevated oxidative stress, increased GGT expression, and effectively eradicated tumors without systemic toxicity.

**Conclusion:** GLOXmp specifically targeted GGT-overexpressing cancer cells and effectively suppressed tumor development through oxidative stress amplification. Given a self-reinforcing strategy for targeted cancer therapy through oxidative stress-mediated tumor cell reprogramming, GLOXmp demonstrates represents a promising advancement in precision nanomedicine.

## Introduction

Over the last few decades, targeted drug delivery systems including micelles, liposomes, and polymeric nanoparticles have been widely explored to enhance the therapeutic efficacy of anticancer drugs, while minimizing their side effects 1. These drug delivery systems are often functionalized with ligands that bind specifically to receptors overexpressed on cancer cells, including folate, integrin-binding peptides, transferrin-binding peptides, and antibodies 2. Although the receptor-mediated targeted drug delivery has been proven in various animal studies, its clinical translation remains challenging due to tumor heterogeneity 3-5. Receptor heterogeneity across tumor tissues, as well as dynamic changes in gene expression within the tumor microenvironment, hamper consistent drug delivery 6. Additionally, some cancers exhibit insufficient receptor expression to support effective targeting of drug delivery systems. Therefore, it is critical to develop a promising strategy for upregulating specific receptors through tumor reprogramming that could maximize the therapeutic outcomes of targeted delivery systems 2,3,5,7.

Compared to normal cells, cancer cells have elevated levels of ROS, which play multiple roles in tumor biology. While moderate ROS levels promote cell proliferation and survival, excessive ROS can cause oxidative stress, damaging proteins and DNA, and inducing apoptotic cell death 8. To counteract the detrimental effects of ROS-mediated oxidative stress, cancer cells are well equipped with antioxidant systems, particularly GSH, the most abundant cellular antioxidant, which helps maintain redox balance 9. This inherent biological difference between cancer and normal cells provides a biological cue for selective cancer therapy, where ROS generators, antioxidant scavengers, or their combination can selectively induce cancer cell death 8,10. When exposed to prooxidants therapeutics, cancer cells adapt by increasing the level of GSH. This process is regulated by GGT, a membrane-bound enzyme that catabolizes extracellular GSH and transfers glutamyl moiety for the synthesis of GSH in cells 11-13. In particular, GGT is overexpressed on the plasma membrane of various cancer cells including lung, prostate, cervical and ovarian cancers 14. Therefore, GGT holds great potential as a target receptor for both diagnostics and therapy of cancer. In addition, GGT is known to have a high affinity to PGA, a biodegradable polymer of glutamic acid (Glu) residues, further supporting its role as a therapeutic target 2,15,16. Based on this, we hypothesized that a short glutamate-based sequence, such as the glutamic acid decapeptide (Glu_10_), representing a defined segment of PGA, could hold GGT-binding ability and serve as an effective cancer targeting ligand.

**Scheme 1:**
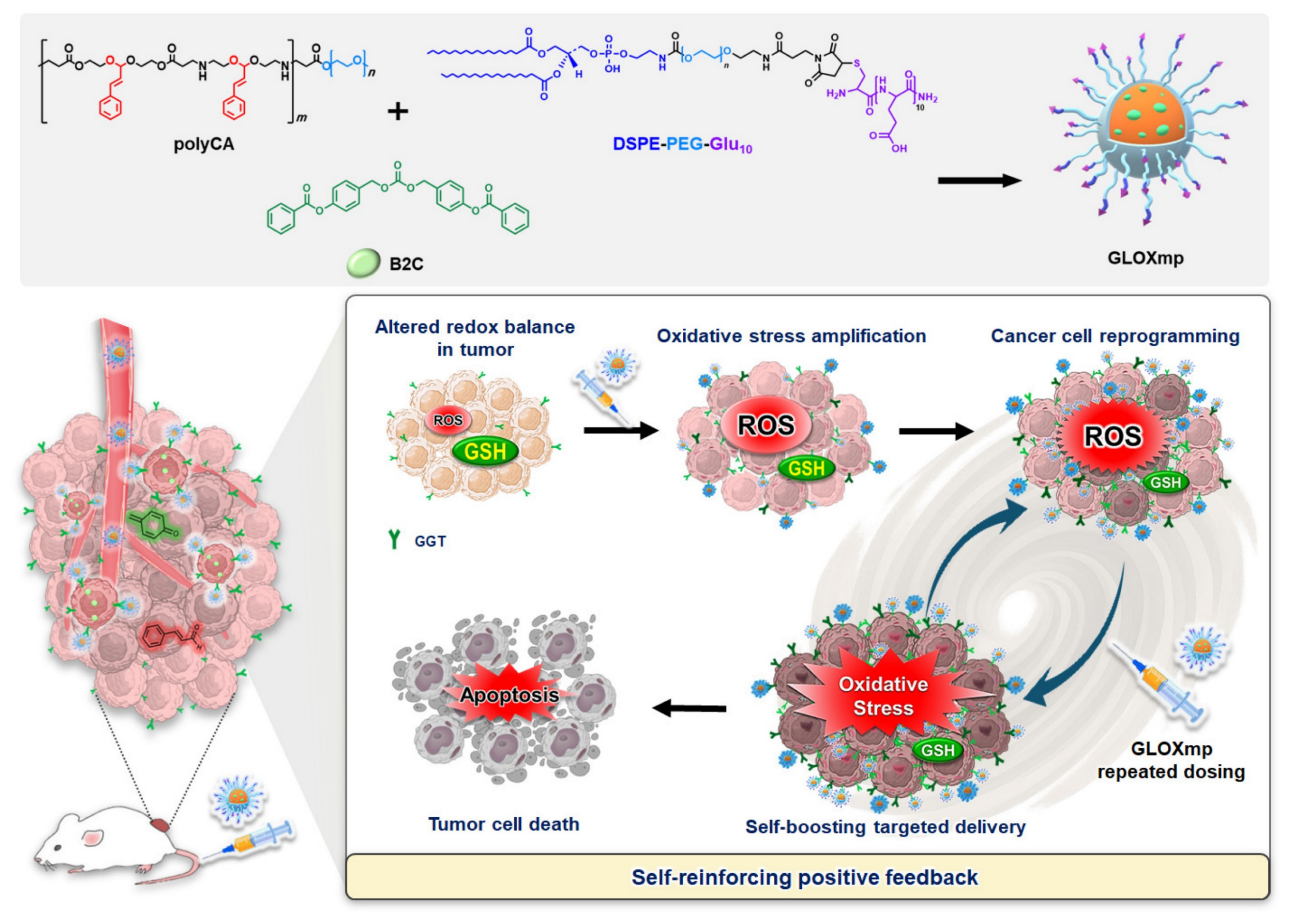
Schematic illustration of the self-boosting targeted anticancer mechanism of GLOXmp. After GGT-mediated accumulation in tumors, GLOXmp simultaneously generates ROS and depletes GSH, leading to amplified oxidative stress. The elevated oxidative stress further induces GGT overexpression, which enhances subsequent tumor accumulation of GLOXmp and establishes a positive feedback loop for effective tumor cell killing.

In this context, we hypothesized that the Glu-decorated prooxidants not only elevate oxidative stress to kill cancer cells preferentially while actively reprogramming them to upregulate GGT expression. This approach would establish self-reinforcing positive feedback: GGT upregulation enhances targeted delivery of subsequent doses in a positive feedback loop, leading to self-boosting targeted anticancer therapy. To demonstrate the concept of the self-boosting targeted anticancer therapy through oxidative stress amplification and cancer cell self-reprogramming, we constructed GLOXmp, a micelle-based nanomedicine comprising ROS-generating polymeric prodrug (polyCA), GSH-depleting agent (B2C), and GGT-binding Glu_10_. polyCA, a biodegradable amphiphilic polymer, forms stable micelles and releases cinnamaldehyde (CA) at only acidic intracellular pH to generate ROS to induce apoptosis 17. As previously reported, B2C is a prooxidant that effectively depletes GSH to kill cancer cells preferentially rather than normal cells 10,18. Glu_10_, incorporated as a targeting moiety in amphiphilic lipopeptide, DSPE (1,2-distearoyl-sn-glycero-3-phosphoethanolamine)-PEG (polyethylene glycol)-Glu_10_ (DSPE-PEG-Glu_10_), facilitates selective binding to GGT-overexpressing cancer cells. polyCA and DSPE-PEG-Glu_10_ could self-assemble to form stable micelles to encapsulate B2C in their hydrophobic interior. We also hypothesized that Glu_10_ exists on their surface and targets GGT-overexpressing cancer cells. GLOXmp is therefore expected to achieve two synergistic outcomes. First, in acidic intracellular environments, GLOXmp releases both ROS-generating CA and GSH-depleting B2C, synergistically amplifying oxidative stress to induce apoptotic cell death. Second, the amplified oxidative stress upregulates GGT expression, enhancing the targeted delivery of subsequent doses of GLOXmp. We anticipate that this self-boosting mechanism provides a positive feedback loop to synergistically enhance anticancer therapeutic efficacy (Scheme 1). Here, we present a comprehensive investigation of potent anticancer effects of GLOXmp using cell culture models and mouse xenograft models.

## Results

### Synthesis and characterization of polyCA

**Figure 1:**
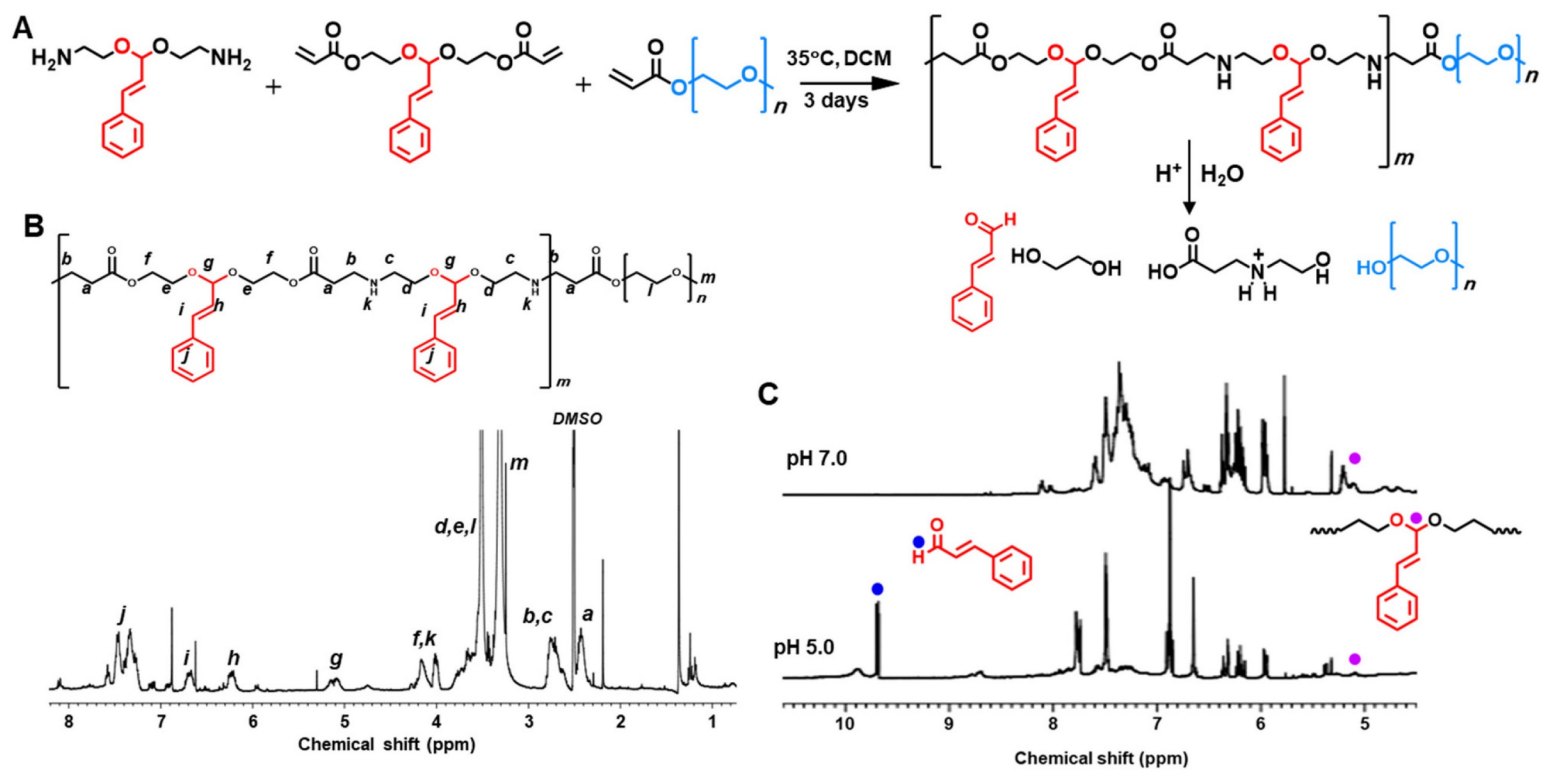
** polyCA as an acid-triggered ROS generating amphiphilic polymer.** (A) A synthetic route of polyCA and its acid-triggered degradation. (B) ^1^H NMR spectrum of polyCA. (C) ^1^H NMR spectra of polyCA treated at pH 7.0 and pH 5.0. polyCA and degrading products were lyophilized and subjected to NMR analysis. Blue dots and purple dots indicate aldehyde proton and acetal proton, respectively.

We first developed polyCA as a major component of GLOXmp which performs dual functions as an oxidative stress amplifier and a carrier of antioxidant scavengers. polyCA was designed to incorporate CA in its hydrophobic backbone via an acetal linkage, enabling controlled release and ROS generation at acidic tumor pH. As shown in Figure 1A, the synthesis of polyCA leveraged the advantages of poly(β-amino ester) such as biocompatibility, excellent chemical tunability, rapid acid-responsiveness, and mild reaction condition 19,20. To allow micelle formation in aqueous environments through self-assembly, polyCA was engineered as an amphiphilic copolymer with a hydrophobic CA-incorporated segment and a hydrophilic PEG segment. For the synthesis of polyCA, CA-incorporated diamine was first synthesized from a two-step reaction (Figure S1). CA reacted with phthalimide and then the phthalimide protection groups were removed under the alkaline condition. CA-incorporated diacrylate was synthesized from the reaction of CA with hydroxyethyl acrylate (Figure S1). In both CA-incorporated diamine and CA-incorporated diacrylate, the CA moiety was incorporated through an acid-labile acetal linkage. Among several acid-labile linkages, we selected acetal because its acid-triggered degradation could liberate an original aldehyde moiety that is a critical part of unsaturated carbonyl Michael pharmacophore of CA. The chemical structure of CA-derivatives was confirmed by NMR (Figure S2).

polyCA was synthesized from Michael addition reaction of CA-incorporated diamine, CA-incorporated diacrylate and PEG-methyl ether acrylate at a molar ratio of 10:9:2. After 3 days of polymerization reaction at 35 ºC, polyCA was obtained as a rubbery solid polymer. The chemical structure of polyCA was verified by NMR (Figure 1B) and its molecular weight was determined to be ~16,800 Da by diffusion-ordered NMR spectroscopy (Figure S3). The presence of characteristic protons such as acetal protons (5.3 ppm) and ethylene protons (2.5 and 3.8 ppm) between amine and ester confirms the successful synthesis of polyCA. As polyCA was expected to release CA through the acid-triggered degradation of acetal linkage, NMR analysis was conducted to investigate acid-responsiveness of polyCA. Hydrolysis of polyCA at pH 5.0 resulted in the disappearance of acetal protons and the emergence of aldehyde protons at ~9.6 ppm (Figure 1C), confirming CA release through acid-triggered hydrolytic degradation. Quantitative analysis revealed that 1 mg of polyCA could release ~ 300 μg of CA. Taking together, the successful synthesis and characterization of polyCA confirm its dual functionality as an acid-cleavable polymeric prodrug of CA. The acid-labile acetal linkage is expected to enable precise, pH-triggered release of CA, preserving its critical Michael acceptor pharmacophore.

### Preparation and characterization of polyCA micelles and GLOXmp

**Figure 2:**
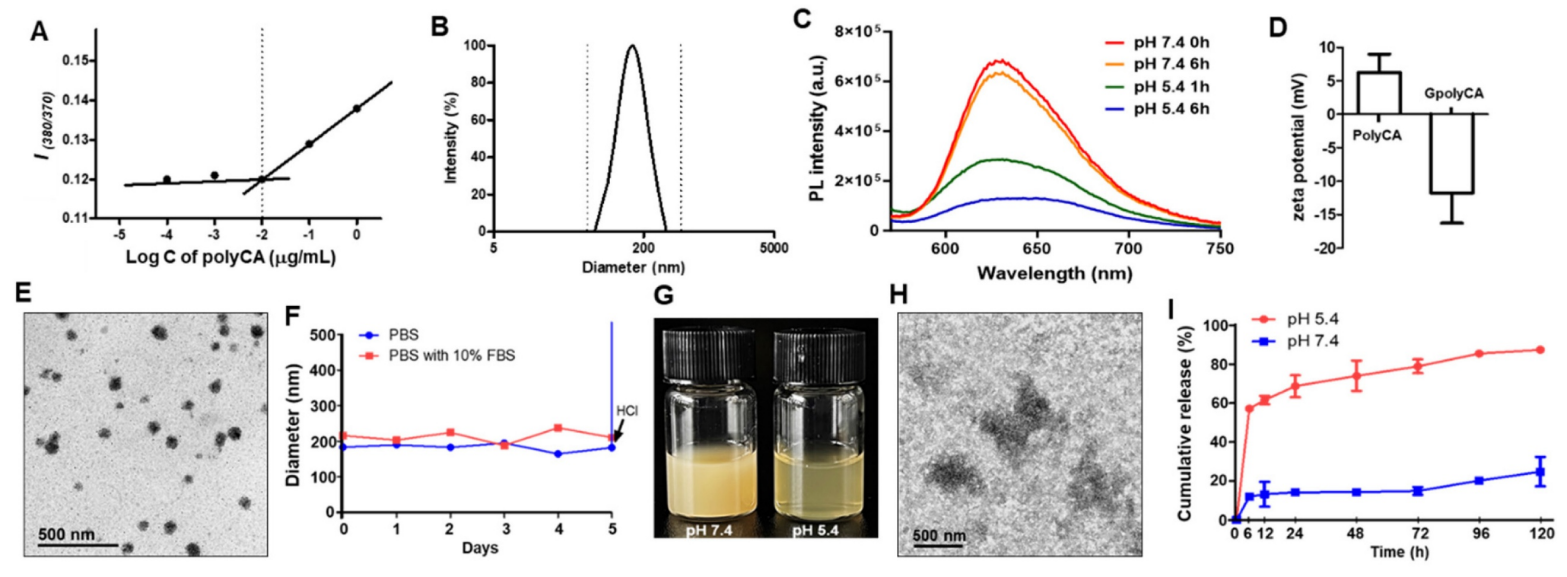
** Characterization of GLOXmp.** (A) Fluorescence intensity ratio of pyrene-loaded micelles as a function polyCA micelle concentration. Critical micelle concentration (CMC) is identified as the point of inflection where fluorescence intensity begins to increase, indicating micelle formation. (B) Size distribution of polyCA micelles determined by dynamic light scattering based on intensity-weighted distribution. Polydispersity index: 0.15. (C) Fluorescence spectra of Nile red-loaded polyCA micelles at different pH. (D) Zeta potential of polyCA and GpolyCA micelles at pH 7.0. Values are mean ± s.d. (n = 3). (E) TEM image of GLOXmp. (F) Colloidal stability of GLOXmp under physiological conditions. (G) Photographs of GLOXmp at pH 7.4 and 5.4. (H) Representative TEM image of GLOXmp after incubation at pH 5.4 for 6 h. (I) Release rate of CA from GLOXmp at different pH. Values are mean ± s.d. (n = 4).

polyCA was designed to exhibit amphiphilic nature, with a CA-containing hydrophobic segment and a hydrophilic PEG segment. Under the aqueous condition, polyCA self-assembled into micelles at concentrations above ~10 μg/mL, with a mean hydrodynamic diameter of ~170 nm (Figure 2A-B). To examine the pH-responsiveness of the micelles, Nile red, a hydrophobic fluorescent dye was used. At pH 7.4, Nile red-loaded polyCA micelles displayed a distinct fluorescence peak at 615 nm, reflecting the presence of a stable, nonpolar environment. However, at pH 5.4, the fluorescence intensity significantly decreased over time, suggesting that polyCA micelles are stable at neutral pH but release their encapsulated payloads under acidic conditions (Figure 2C). This acid-triggered disruption of GLOXmp is attributed to the rapid protonation of secondary amine in the polyCA backbone with pKa values ranging from 6.5 to 7.5, indicating a hydrophobic-hydrophilic transition 17,19. The amphiphilic lipopeptide, DSPE-PEG-Glu_10_ was also used, featuring a hydrophobic DSPE segment and a hydrophilic PEG-Glu_10_ segment. A mixture of polyCA and DSPE-PEG-Glu_10_ self-assembled under aqueous conditions to form Glu_10_-decorated polyCA (GpolyCA) micelles. These GpolyCA micelles maintained a hydrodynamic diameter similar to polyCA micelles and were spherical in morphology. The hydrophobic core was likely formed by both DSPE and CA-incorporated segments, while PEG and Glu_10_ formed their hydrophilic corona. The presence of Glu_10_ on the surface of GpolyCA micelles could be indirectly confirmed by the zeta potential measurements. polyCA micelles exhibited slightly positive surface charge of + 7.4 mV at neutral pH, whereas GpolyCA micelles showed a negative surface charge, -11.1 mV (Figure 2D), because of the abundant carboxylic groups in decapeptide Glu_10_. These observations suggest that Glu_10_ is present on the surface of GpolyCA micelles.

GSH-depleting B2C was synthesized via a three-step reaction and its chemical structure was verified by NMR (Figure S4). B2C degrades in an esterase-triggered manner to release quinone methide (QM) intermediate, which effectively alkylates GSH 10. As an oxidative stress-amplifier, GLOXmp was formulated by encapsulating B2C within the self-assemblies of polyCA and DSPE-PEG-Glu_10_ (Scheme 1). The loading content of B2C was 10 wt% and the efficiency was 83%. GLOXmp exhibited spherical morphology with a mean diameter of ~180 nm (Figure 2E). The stability of GLOXmp was assessed under physiological conditions containing 10 wt% serum protein. GLOXmp showed negligible changes in the hydrodynamic diameter over 5 days, confirming their stability in neutral environments (Figure 2F). However, when pH was reduced to 5.0 using HCl, GLOXmp exhibited a substantial increase in diameter and became transparent (Figure 2G). TEM imaging revealed complete loss of spherical morphology and colloidal stability (Figure 2H), indicating that acidic pH induces the disruption of micellar formation. To further confirm the acid-triggered release mechanism, we investigated CA release profiles from GLOXmp. At neutral pH, less than 20% CA was released within 24 h (Figure 2I). In contrast, at pH 5.4, a majority of CA was released within 24 h. These observations demonstrate that GLOXmp dissociates in response to acidic pH, effectively releasing both CA and B2C.

### Cellular uptake and oxidative stress amplification of GLOXmp

We first evaluated cellular uptake and intracellular trafficking of GLOXmp because these properties are critical determinants of intracellular drug delivery. For confocal fluorescence microscopy, GpolyCA micelles, the nanocarrier component of GLOXmp, were loaded with IR780 as a fluorescent probe. As shown in Figure S5, GpolyCA micelles were efficiently internalized by SW620 cancer cells (human colon cancer cell line), as evidenced by a time-dependent increase in intracellular fluorescence. Notably, cellular uptake was significantly reduced at 4°C, confirming the cellular uptake occurs via energy-dependent endocytosis. To further investigate the intracellular trafficking, cells were co-stained with LysoTracker Green to visualize endosomes. Colocalization of GpolyCA micelles with LysoTracker signal produced a distinct yellow fluorescence at early time points, indicating their endosomal entrapment. From 3 h post-incubation, strong green fluorescence disappeared, suggesting the successful endosomal escape. This escape is likely facilitated by two cooperative mechanisms: (i) the proton sponge effect of the secondary amine groups in the polyCA backbone 21, and (ii) the acid-triggered degradation of polyCA into numerous small molecules. The latter likely increases the osmotic pressure, which can lead to endosomal membrane rupture and promote cytosolic release of the therapeutic payload (ROS-generating CA and GSH depleting B2C) 22.

**Figure 3:**
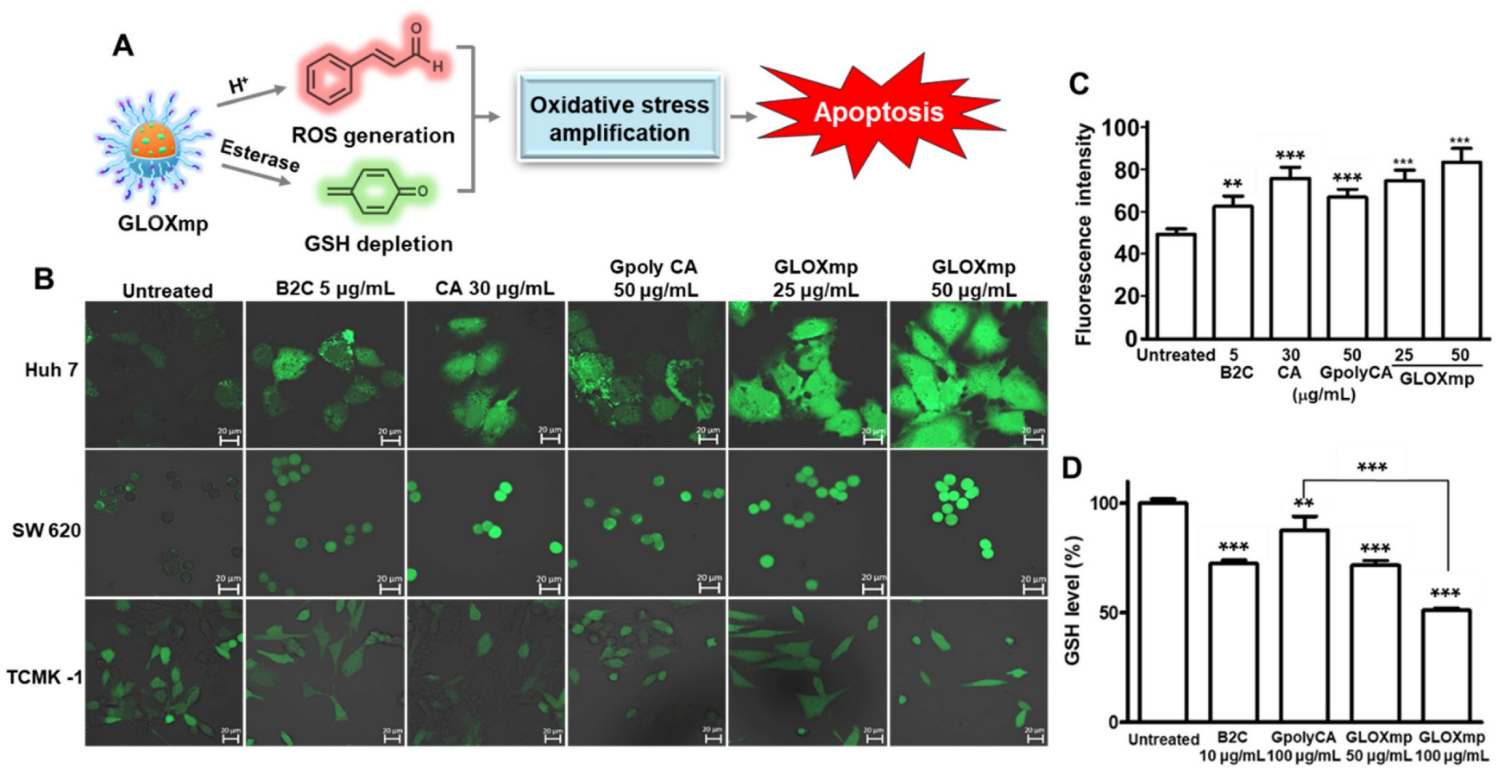
GLOXmp as an oxidative stress nanoamplifier. (A) A schematic of anticancer actions of oxidative stress amplifying GLOXmp. (B) Fluorescence images of cells treated with B2C, polyCA or GLOXmp for 12 h. Cells were stained with DCFH-DA. (C) Quantification of the intracellular level of ROS in SW620 cells. (D) The reduction of GSH levels in SW620 cells treated with GLOXmp. Values are mean ± s.d. (n = 4). *** p < 0.001, ** p < 0.01 relative to untreated cells.

GLOXmp was designed as a prooxidant nanomedicine that simultaneously generates ROS and depletes antioxidant GSH, significantly amplifying oxidative stress and killing cancer cells (Figure 3A). As polyCA is the primary ROS-generating component of GLOXmp, we first assessed the ability of polyCA micelles to generate ROS in cancer cells using DCFH-DA (dichlorodihydrofluorescein-diacetate) as a ROS probe which fluoresces upon oxidation. polyCA, synthesized as a polymeric prodrug of CA, induced time-dependent ROS generation in SW620 cells, as evidenced by the gradual increase in green fluorescence signal (Figure S6). We next investigated the ROS-generating ability of GLOXmp using various cells, SW620 cells, Huh7 cells (human hepatocellular carcinoma cell line) and TCMK-1 cells (murine kidney cell line) (Figure 3B-C and S7). As expected, polyCA micelles generated ROS in a concentration-dependent manner, like CA. B2C also elevated the level of ROS because of its ability to deplete antioxidant GSH, thereby reducing the cellular antioxidant capacity. GLOXmp exhibited significantly higher ROS accumulation than equivalent doses of GpolyCA, B2C, or CA, demonstrating a synergistic effect of the combined components. Importantly, GLOXmp (50 µg/mL) elevated the level of ROS in cancer cells, with negligible induction observed in normal TCMK-1cells (Figure S7B). We also evaluated the ability of GLOXmp to scavenge intracellular GSH in various cells. After 4 h of incubation with polyCA, B2C or GLOXmp, cells remained viable, allowing the assessment of intracellular GSH levels (Figures 3D and S8). Both GLOXmp and the molar-equivalent B2C significantly reduced the intracellular GSH. This suggests sufficient release of B2C from GLOXmp, consistent with the observed disruption of polyCA micelles within 6 h (Figure 2I). These findings demonstrate that GLOXmp effectively amplifies oxidative stress through a synergistic combination of ROS generation and GSH depletion, selectively targeting cancer cells while sparing normal cells. These findings suggest that GLOXmp exerts selective and potent anticancer actions and hold potential for overcoming limitations of conventional oxidative stress-based therapy.

### *In vitro* anticancer activities of GLOXmp

**Figure 4:**
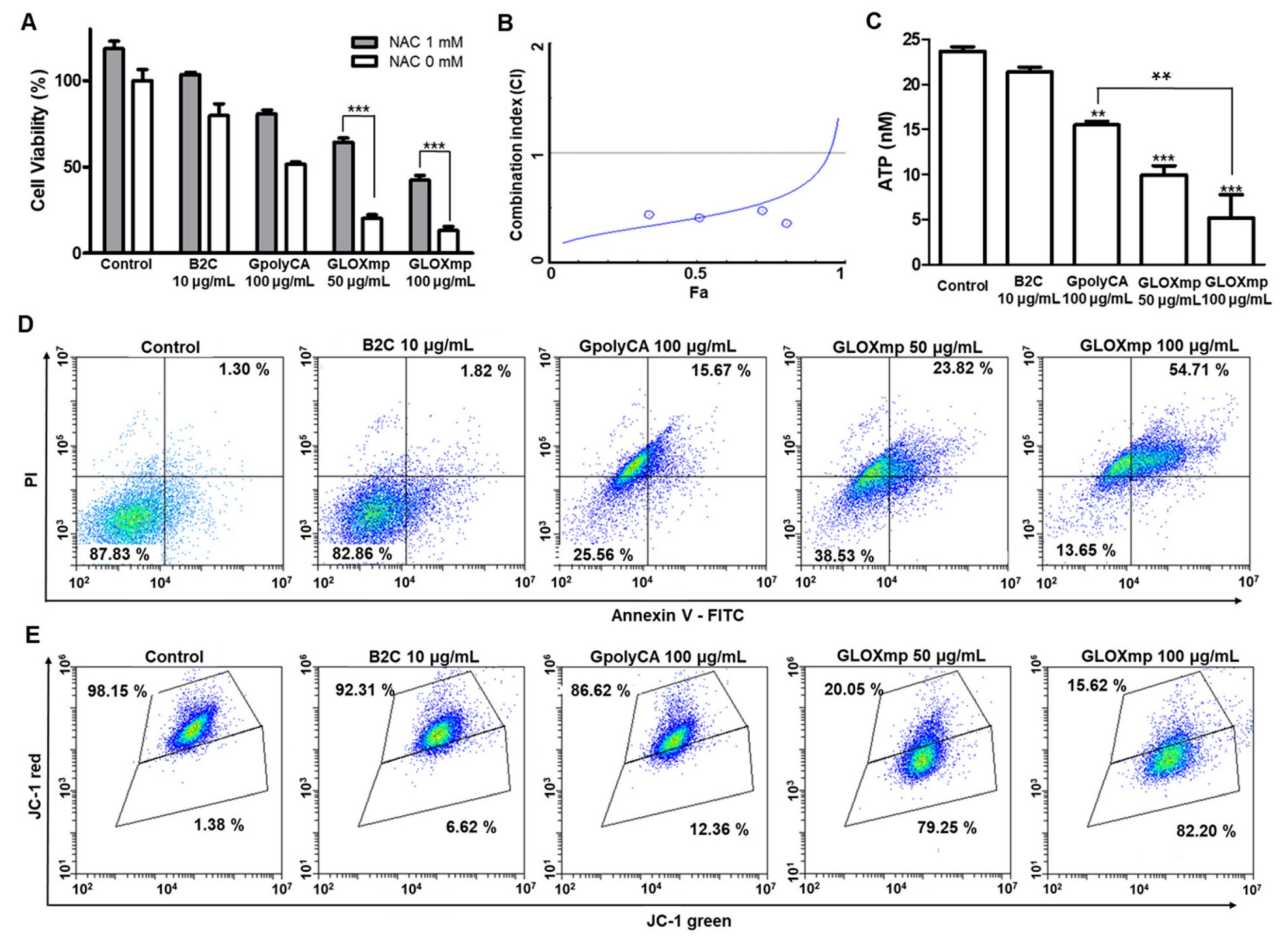
** Anticancer activities of GLOXmp.** (A) Viability of SW620 cells treated with GLOXmp in the presence or absence of NAC. (B) CI versus affected cell-fraction (Fa) curves for the combination of polyCA micelles with B2C. (C) The intracellular level of ATP in SW620 cells treated with B2C, GpolyCA or GLOXmp. Values are mean ± s.d. (n = 4). *** p < 0.001, ** p < 0.01 relative to untreated cells. Flow cytometric analysis of SW620 cells stained with (D) Annexin V-FITC and (E) JC-1 following the treatment with GpolyCA, B2C or GLOXmp.

The cytotoxicity of GLOXmp was evaluated by the conventional MTT (3-(4,5-dimethylthiazol-2-yl)-2,5-diphenyltetrazolium bromide) assay using cancer cells and normal cells. GLOXmp displayed significant cytotoxicity against various cancer cell lines (SW620, Huh7, MCF-7 and A549), concentration dependently (Figure 4A and S9A-C). For example, the viability of SW620 cells decreased to 15% at 100 μg/mL of GLOXmp. Importantly, GLOXmp demonstrated significantly greater cytotoxicity than equivalent doses of B2C or GpolyCA micelles alone, suggesting the benefit of combining ROS generation (polyCA) and GSH depletion (B2C) in a single formulation. N-acetyl cysteine (NAC), an antioxidant, significantly suppressed the cytotoxicity of GLOXmp, confirming that oxidative stress is the primary driver of its anticancer activity. To quantitatively assess the synergistic interaction between polyCA micelles and B2C, we performed an additional MTT assay and determined the combination index (CI) using the Chou-Talalay method. polyCA micelles and B2C showed strong synergism, as evidenced by the CI values significantly lower than 1.0 (Figure 4B). This result confirms that the concurrent delivery of ROS generators and GSH scavengers amplifies oxidative stress beyond additive effects, leading to enhanced anticancer efficacy. Notably, GLOXmp exhibited markedly lower cytotoxicity against normal cells (RAW264.7 and TCMK-1) (Figure S9D-E). The cancer cell preferential cytotoxicity of GLOXmp can be attributed to the altered redox balance in cancer cells compared to the well-maintained redox balance in normal cells 8,23-25. These findings also highlight the therapeutic selectivity of GLOXmp.

As cancer cells accelerate mitochondrial ATP production and have higher level of intracellular ATP than normal cells, high mitochondrial ATP production is considered a new therapeutic target for cancer treatment 26. We therefore investigated the effects of GLOXmp on the level of intracellular ATP. GLOXmp significantly reduced the ATP levels in both SW620 and Huh7 cells compared to equivalent doses of GpolyCA micelles or B2C (Figure 4C and S10). To assess whether GLOXmp induces apoptosis via ROS accumulation, flow cytometric analysis was performed using FITC-Annexin V as an apoptosis marker and propidium iodide (PI) as a viability marker. Both B2C and GpolyCA micelles induced apoptosis, as supported by the increase in the population of the upper right quadrant (Figure 4D and S11A). GLOXmp induced similar or greater levels of apoptosis. In particular, the group treated with 100 μg/mL GLOXmp showed a marked shift of the cell population to the upper right quadrant, reflecting an increased population of Annexin V^+^/PI^+^ cells, indicative of late apoptosis or secondary necrosis. This dose-dependent shift suggests that higher concentrations of GLOXmp trigger oxidative damage to a greater extent, leading to membrane damage and apoptosis. The results also demonstrate the synergistic effects of its components, B2C and polyCA. We next examined the effects of GLOXmp on the mitochondrial membrane potential, a hallmark of early apoptosis triggered by ROS 10. GLOXmp treatment reduced the red-to-green fluorescence intensity, suggesting a concentration-dependent reduction in mitochondrial membrane potential (Figure 4E and S11B). The extent of mitochondrial membrane disruption caused by GLOXmp was significantly greater than those of equivalent B2C or GpolyCA micelles. Taking together, GLOXmp effectively induces apoptosis through ROS-mediated oxidative stress, mitochondrial disruption, and ATP depletion. These findings also underscore the potential of GLOXmp as a highly selective and effective anticancer nanomedicine that exploits cancer cells' vulnerabilities in redox homeostasis and mitochondrial function.

### GGT-mediated cancer cell targeting of GLOXmp *in vitro*

**Figure 5:**
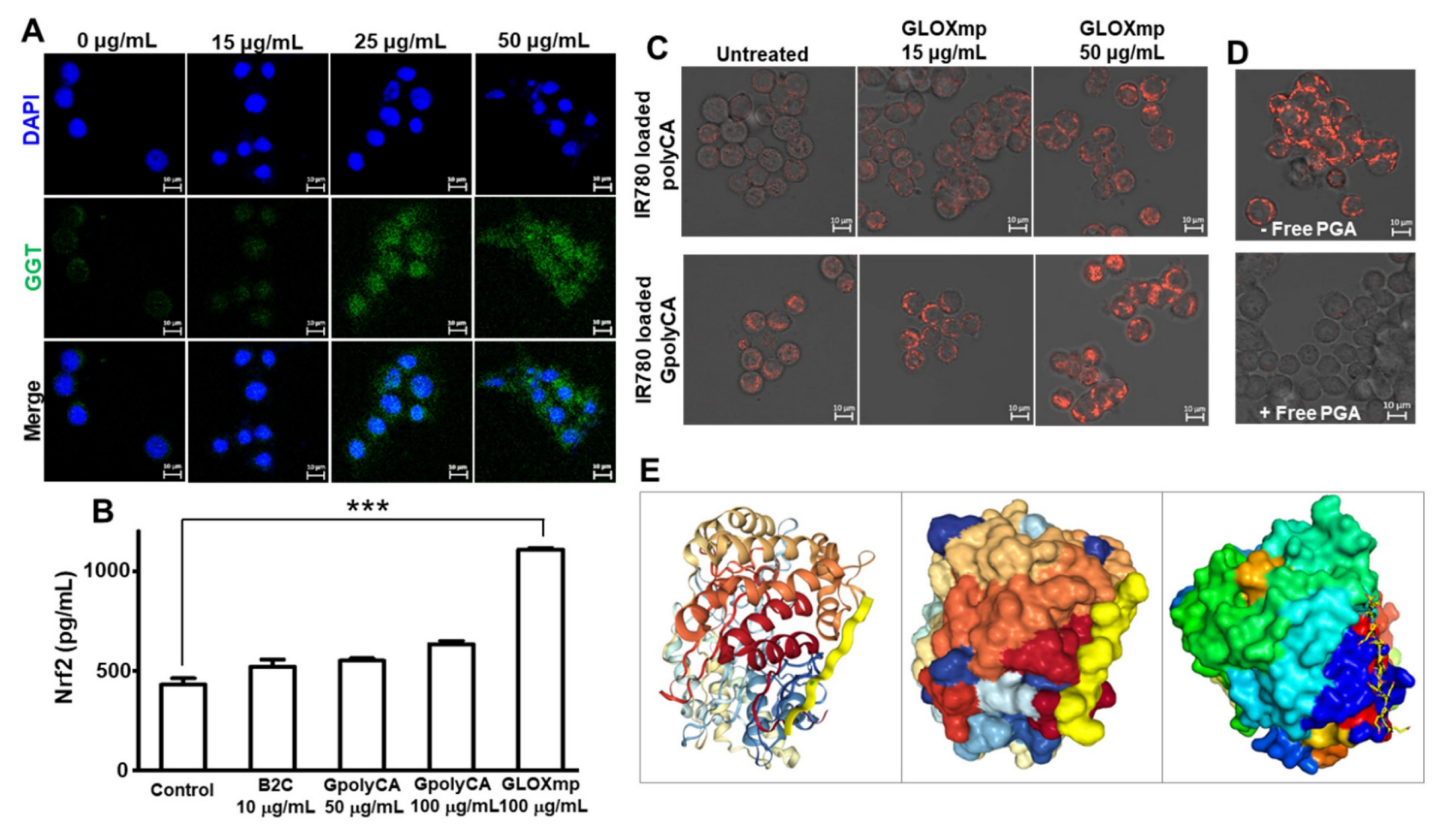
** GGT-dependent cellular uptake of GLOXmp.** (A) Fluorescence images of SW620 cells showing the GLOXmp-induced GGT expression. (B) The level of nuclear Nrf2 in SW620 cells treated with various concentration of GLOXmp. Values are mean ± s.d. (n = 4). ***p < 0.001. (C) Fluorescence images of SW620 cells incubated with fluorescent IR780-loaded GpolyCA micelles. Images were made at 0.5 h-post incubation. (D) Fluorescence images of SW620 cells incubated with IR780-loaded GpolyCA micelles in the presence (+) or absence (-) of free PGA. Images were made at 6 h-post incubation. (E) Molecular docking of Glu_10_ to GGT simulated in a peptide-protein docking platform (HPEPDOCK 2.0 server).

Since GLOXmp was developed as GGT-targeting anticancer therapeutics, we examined the cell membrane level of GGT on various cells. Cancer cell lines (SW620 and Huh7 cells) exhibited a higher cell membrane level of GGT compared to RAW264.7 cells and HEK293 cells (Figure S12), consistent with previous reports 27,28. We next investigated whether oxidative stress amplifying GLOXmp enhances the expression of GGT on the cancer cell membrane because GGT is known to be upregulated by oxidative stress 29. Cells were treated with GLOXmp for 12 h, stained with a fluorescent anti GGT-antibody and observed under a confocal fluorescence microscope. As shown in Figure 5A, GLOXmp treatment significantly enhanced the GGT expression on the membrane of SW620 cells, in a concentration-dependent manner. This finding demonstrates that oxidative stress amplifying GLOXmp actively reprograms cancer cells to upregulate membrane-bound GGT, creating a positive feedback loop for self-boosting targeted delivery. To further explore the underlying molecular mechanism of this upregulation, we examined the nuclear levels of nuclear factor E2-related factor 2 (Nrf2), a redox-sensitive transcription factor known to regulate antioxidant response elements, including GGT transcription (Figure 5B). ELISA analysis revealed a dose-dependent increase in nuclear Nrf2 following GLOXmp treatment, indicating that GLOXmp induces ROS generation to activate the Nrf2 signaling pathway.

To further investigate the role of GGT in cancer cell uptake, IR780 was employed as a fluorescent probe. SW620 cells were pre-treated with 15 μg/mL of GLOXmp for 12 h to induce GGT upregulation, followed by incubation with either fluorescent GpolyCA micelles or polyCA micelles (Figure 5C). At 30 min post-incubation, cells treated with GpolyCA micelles exhibited significantly higher fluorescence compared to those treated with polyCA micelles, indicating the critical role of Glu_10_ in facilitating cellular uptake. Furthermore, the cellular uptake of fluorescent GpolyCA micelles in GLOXmp-pretreated cells was significantly reduced in the presence of free PGA which competes with GpolyCA micelles for GGT binding (Figure 5D). These findings demonstrate that Glu_10_ on the surface of GpolyCA micelles acts as a GGT targeting ligand, accelerating cellular uptake in a GGT-dependent manner. In addition, the result of molecular docking simulation of Glu_10_ with GGT revealed favorable binding confirmation, supporting its potential role in GGT recognition (Figure 5E). These results reinforce the hypothesis that Glu_10_, a short glutamate-rich sequence mimicking the structural and functional features of PGA, exhibits sufficient GGT-binding affinity to mediate targeted uptake. Additionally, GLOXmp reprograms cancer cells to overexpress GGT, thereby enhancing GGT-dependent endocytosis of Glu_10_-coated nanomaterials. This dual functionality supports the potential of GLOXmp as a self-boosting targeted anticancer nanomedicine that selectively exploits the vulnerabilities of cancer cells. It can be also noted that GLOXmp not only leverages existing GGT expression but also induces GGT regulation, creating a self-reinforcing positive feedback loop that enhances their selective uptake and therapeutic efficacy in cancer cells.

### Self-boosting targeted anticancer therapy of GLOXmp *in vivo*

**Figure 6:**
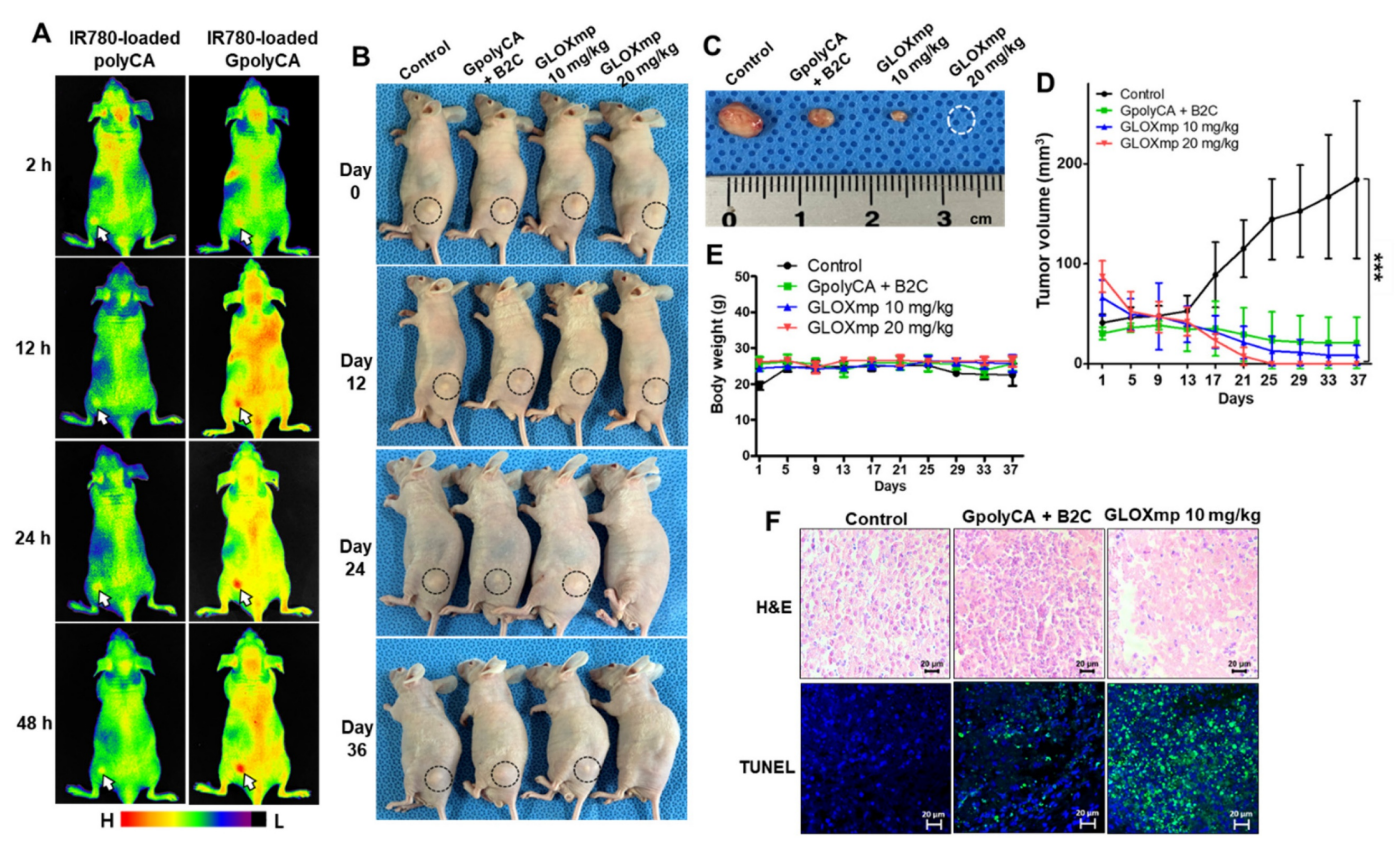
***In vivo* anticancer activity of GLOXmp.** (A) Fluorescence images of tumor-bearing mice injected with fluorescent polyCA micelles or GpolyCA micelles. (B) Photographs of tumor-bearing mice during the treatment with various formulations. (C) Photographs of tumors excised on day 37. Changes of (D) tumor volume and (E) body weight during the treatment. Values are mean ± s.d. (n = 4). ***p <0.001. (F) Micrographs of tumor tissues stained with H&E (top) and TUNEL (bottom).

The key features of GLOXmp, including cancer cell preferential cytotoxicity and its ability to biochemically reprogram cancer cells to upregulate GGT expression, motivated us to investigate their therapeutic efficacy in vivo using a xenograft mouse model of SW620 human colon cancer cells. To assess the biodistribution of GLOXmp, fluorescent IR780-loaded GpolyCA micelles decorated with Glu_10_ were administered to tumor-bearing mice. As shown in Figure 6A, strong fluorescence signals were observed at the tumor site as early as 2 h post-injection, with the intensity increasing over time. Notably, tumors in mice treated with fluorescent GpolyCA micelles displayed significantly higher fluorescence than those treated with fluorescent polyCA micelles lacking Glu_10_, demonstrating that Glu_10_ confers enhanced tumor targeting. This tumor preferential accumulation of GpolyCA micelles can be ascribed to Glu_10_'s structural similarity to PGA, which binds GGT. Furthermore, this targeting may be facilitated by Glu_10_'s ability to reduce non-specific protein binding and evade immune recognition, promoting prolonged systemic circulation and enhanced tumor penetration 2,30. For imaging-based biodistribution study, we conducted a pharmacokinetic analysis by evaluating the fluorescence intensity of IR780-loaded GpolyCA micelles in blood after systemic injection (Figure S13). GpolyCA micelles showed significantly higher fluorescence levels and longer circulation times compared to polyCA micelles, confirming that Glu_10_ enhances *in vivo* stability and systemic circulation, a key feature contributing to the improved tumor targeting of GLOXmp.

To assess the anticancer therapeutic efficacy of GLOXmp, tumor-bearing mice were randomized into four groups when the average tumor volume reached ~50 mm^3^. Tumor-bearing mice were injected with the mixture of B2C and GpolyCA micelles (20 mg/kg) or GLOXmp (10 and 20 mg/kg) every 3 days for a total of 10 doses. Tumor growth in the untreated group progressed steadily over 36 days. Both GpolyCA micelles and the B2C/GpolyCA mixture inhibited tumor growth. However, GLOXmp demonstrated significantly stronger dose-dependent anticancer effects (Figure 6B-D and S14A). In particular, 20 mg/kg of GLOXmp almost completely eradicated tumors, leaving no discernable remnants. Importantly, no significant changes in body weight were observed during the 36 days of observation period (Figure 6E and S14B).

To further assess the anticancer activity of GLOXmp, tumor tissues were excised and subjected to histological analysis. Tumors of the group treated with 20 mg/kg of GLOXmp were almost completely eradicated and therefore could not be subjected to the histological study (Figure 6F). Tumors treated with GLOXmp (10 mg/kg) showed significant morphological changes including nuclear shrinkage and fragmentation, membrane damages and disrupted cellular architecture. Compared to other groups, GLOXmp-treated tumors also showed a significantly higher DHE (dihydroethidium) fluorescence (Figure S15), indicative of increased ROS accumulation. Additionally, terminal deoxynucleotidyl transferase dUTP nick end labeling (TUNEL) staining revealed a greater number of apoptotic cells in GLOXmp-treated tumors compared to other groups. These observations demonstrate that GLOXmp amplifies oxidative stress through the concurrent delivery of GSH-depleting B2C and ROS-generating CA. The higher therapeutic efficacy of GLOXmp over the equivalent mixture of B2C and GpolyCA micelles could be explained by the rationale that GLOXmp accumulates in tumors preferentially and delivers both GSH-depleting QM and ROS-generating CA concurrently to cancer cells, synergistically amplifying oxidative stress to kill cancer cells. This synergistically amplified oxidative stress overwhelms cancer cells' antioxidant defenses to induce apoptosis, effectively suppressing tumor growth. Interestingly, GLOXmp induced no abnormal histological changes in liver and kidney (Figure S16). These observations confirm that GLOXmp selectively induces oxidative stress in cancer cells without harming healthy tissues, suggesting their potential as a safe and effective anticancer nanomedicine.

### Self-boosting GGT-mediated tumor targeting of GLOXmp *in vivo*

**Figure 7:**
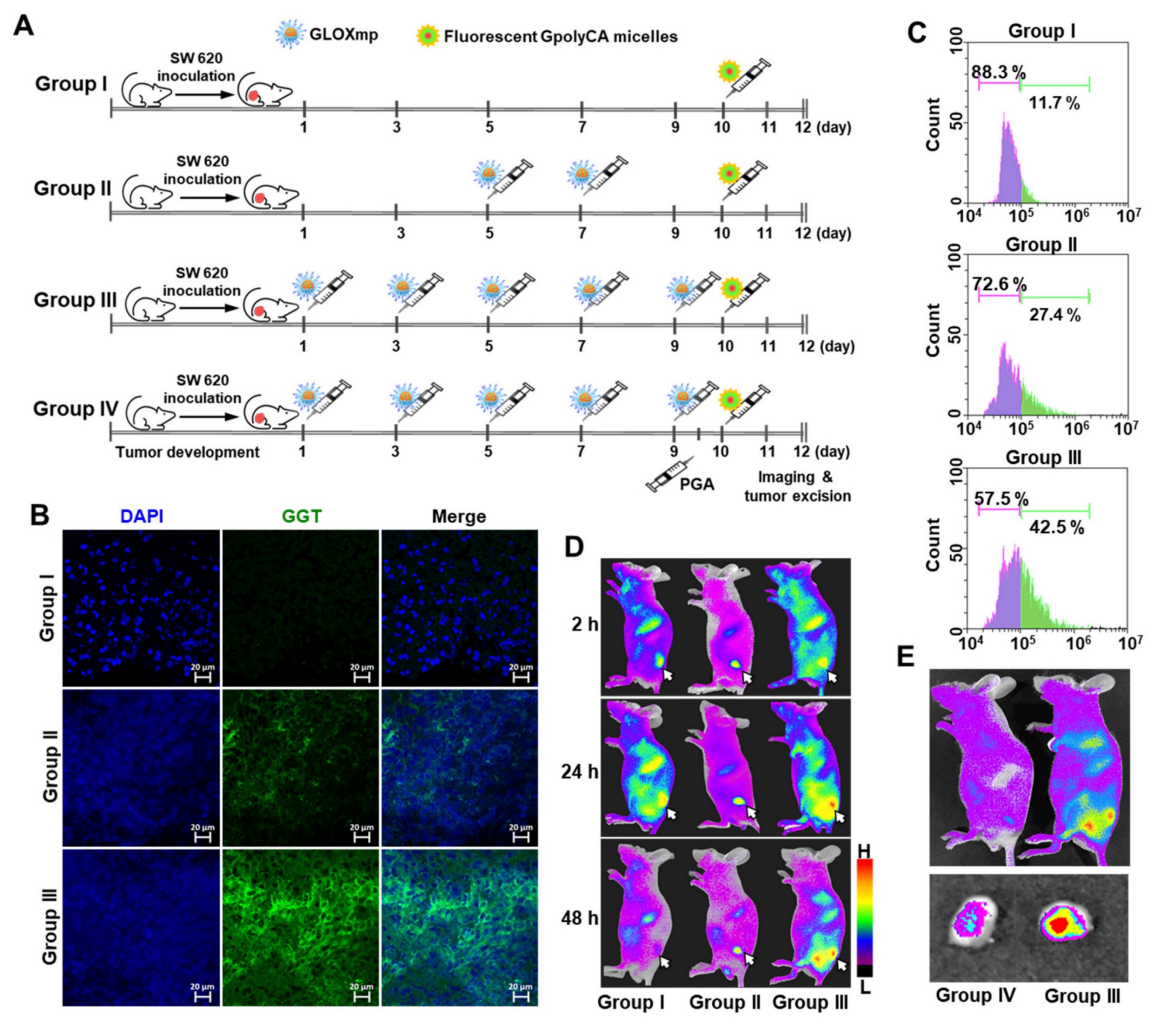
** Self-boosting targeted delivery of GLOXmp.** (A) An experimental timeline of animal study. (B) Tumor tissues stained with anti-GGT antibody. (C) Flow cytometric analysis of intratumoral level of GGT after different dosing of GLOXmp. (D) Fluorescence images of tumor-bearing mice (Groups I, II, III) injected with fluorescent GpolyCA micelles. The arrows indicate the tumor site. (E) Fluorescence images of tumor-bearing mice injected with fluorescent GpolyCA micelles in the presence (Group IV) or absence (Group III) of free PGA pretreatment. The bottom is the fluorescence images of excised tumors.

To test the hypothesis that repeated dosing of GLOXmp induces self-boosting, receptor-mediated tumor targeting via GGT upregulation, we investigated whether GLOXmp treatment enhances the GGT expression in a xenograft mouse model. The dose of GLOXmp was limited to 5 mg/kg because doses higher than 10 mg/kg led to significant tumor regression, confounding the assessment of self-boosting effects. When tumor reached approximately ~50 mm^3^, GLOXmp (5 mg/kg) was intravenously injected into mice every 2 days, and tumors were harvested on day 12 to assess GGT expression (Figure 7A). Group III treated with 5 doses of GLOXmp showed the slightly smaller tumors than the untreated Group I. Tumor tissues stained with anti-GGT antibody labeled with Alexa Fluor showed a dose-dependent increase in GGT expression, with the highest expression observed in Group III (Figure 7B and S17). Flow cytometry analysis confirmed that Group III exhibited a significantly larger population of GGT-positive cells compared to the untreated Group I and Group II given 2 doses of GLOXmp (Figure 7C). These observations demonstrate that besides ROS accumulation, GLOXmp biochemically reprograms tumor cells to overexpress GGT, a critical receptor for targeted delivery.

Fluorescence imaging of tumor-bearing mice was also performed to further validate our hypothesis that repeated GLOXmp dosing promotes tumor targeting in a self-boosting manner, enabling self-receptor modulating targeted delivery. As illustrated in Figure 7A, tumor-bearing mice were treated with 2 or 5 doses of GLOXmp (5 mg/kg), followed by intravenous injection of fluorescent GpolyCA micelles one day after the final dose. Tumors in GLOXmp-treated groups (II and III) showed significantly higher fluorescence intensity compared to the untreated Group I, with the highest fluorescence in tumors from the 5-dose GLOXmp group (III) (Figure 7D). These results suggest that repeated dosing of GLOXmp enhances tumor accumulation through GGT-mediated targeting of subsequent doses. More importantly, pretreatment with free PGA inhibited the tumor accumulation of fluorescent GpolyCA micelles. Group IV showed significantly lower tumor fluorescence than group III (Figure 7E), confirming that Glu_10_ on the surface of GLOXmp plays an essential role in GGT-mediated tumor targeting. Taking together, these observations demonstrate that GLOXmp induces receptor-mediated targeting mechanism in a self-boosting manner. Repeated dosing of GLOXmp reprograms tumor cells to overexpress GGT, which in turn enhances accumulation of GLOXmp in tumors and creates a self-reinforcing positive feedback loop, realizing self-boosting targeted therapy and fully maximizes therapeutic efficacy. The highly potent anticancer action of GLOXmp can be explained by this self-boosting mechanism.

### Biosafety of GLOXmp

GLOXmp was administered into healthy mice at a dose of 10 mg/kg for every two days for a total of 8 doses. Blood and major organs (heart, lung, spleen, kidney, and liver) were collected on day 16 for blood chemistry assay and histological analysis. Histological examination revealed that GLOXmp induced no discernible pathological changes in major organs (Figure S18). Additionally, the serum levels of aspartate aminotransferase (AST), alanine aminotransferase (ALT), creatinine and blood urine nitrogen (BUN) were comparable to those in the control group. These results demonstrate that at least at a therapeutic dose, GLOXmp possesses an acceptable biosafety profile.

## Discussion

Targeting cancer cells through receptor-mediated delivery has been hampered by tumor heterogeneity arising from genomic instability and the diverse tumor microenvironment 31,32. One of feasible approaches to overcome the tumor heterogeneity involves active reprogramming of cancer cells to upregulate the expression of specific target receptors. The present study on GLOXmp was inspired by the previously published study showing that ROS reprogram cancer cells to express high cell membrane level of GGT as a target receptor and lead to enhanced delivery of PGA-coated nanomaterials 2. In earlier work, ROS generation for GGT upregulation in the tumor site was induced by the local injection of methylene blue. The elevated level of GGT in tumors markedly enhanced the tumor targeted delivery of phototherapeutic agents, γPGA-interlinked gold nanoclusters that ablate tumors completely with near infrared laser irradiation. Animal studies using xenograft mouse models revealed that methylene blue biochemically reprograms cancer cells to actively modulate the GGT-mediated targeted delivery of γPGA-coated phototherapeutics. However, this strategy for tumor cell reprogramming may be limited to clinical translation because the intratumoral injection of methylene blue is not applicable in clinical practices. However, GLOXmp integrates both functions - ROS generation for GGT upregulation and therapeutic action - into a single nanoformulation, offering a transformative solution for receptor-mediated therapy. Systemically administered GLOXmp amplifies oxidative stress to induce cancer cell death while simultaneously upregulating GGT. This dual functionality effectively enabled a positive feedback loop. The initial dose of GLOXmp selectively targets tumor cells with existing GGT expression and elevates oxidative stress to induce GGT upregulation. The increased GGT expression allows the subsequent doses to target tumor selectively. This self-boosting mechanism allows enhanced tumor targeting to improve therapeutic efficacy as demonstrated in Figures 6-7. The concept of self-boosting targeted therapy using a single nanoformulation, GLOXmp represents a novel paradigm in drug delivery.

To facilitate selective targeting, GLOXmp was decorated with a decapeptide of glutamic acid, Glu_10_, as a GGT-targeting ligand. Because of its structural similarity to PGA, we hypothesized that Glu_10_ holds GGT-binding capacity. To our knowledge, this is the first demonstration of Glu_10_ as a targeting ligand for GGT-mediated delivery, although further studies are needed to validate its targeting efficiency and potential in broader therapeutic contexts. The therapeutic efficacy of GLOXmp is greatly attributed to the synergy between GGT-targeting Glu_10_ and ROS-amplifying polyCA micelles loaded with the GSH scavenging B2C. Early GLOXmp doses reprogram the tumor environment by increasing GGT expression, facilitating the targeting of subsequent doses. This self-reinforcing positive feedback loop provides a promising strategy to overcome the limitations of the conventional receptor-mediated therapy, such as receptor heterogeneity and low expression density.

Furthermore, the self-boosting concept demonstrated by GLOXmp could also be broadly adapted to other ROS nanogenerators 17,33-36, including gold nanoparticles, titanium dioxide or CA derivatives, combined with receptor-specific surface modifications. The self-boosting concept could also be broadly applicable to other ROS nanogenerators, including gold nanoparticles, titanium dioxide or CA derivatives, combined with receptor-specific surface modifications. To our knowledge, GLOXmp represents the first therapeutic platform to achieve self-receptor modulation for self-boosting targeted therapy. However, additional studies are needed to evaluate their efficacy across various tumor models, particularly those with low initial GGT levels or high oxidative stress resistance. While the current study demonstrated promising biocompatibility and no acute toxicity, further investigation is required to assess the long-term safety profile, determine the maximum tolerable dose, and compare therapeutic performance with conventional chemotherapeutics. The innovative self-boosting mechanism introduced by GLOXmp provides a powerful strategy to overcome tumor heterogeneity and maximize the precision and potency of cancer nanotherapeutics in clinical applications.

## Conclusions

We developed GLOXmp to establish a feasible strategy of self-boosting targeted anticancer therapy through cancer cell self-reprogramming. GLOXmp was formulated by self-assembly of acid-triggerable and ROS-generating amphiphilic polyCA, DSPE-PEG-Glu_10_ and GSH-depleting B2C under aqueous conditions. GLOXmp was spherical micelles with a mean diameter of ~180 nm. GLOXmp could generate ROS and deplete GSH concurrently, significantly amplifying oxidative stress, inducing mitochondrial damage and depleting intracellular ATP to effectively kill cancer cells. In addition, GLOXmp induced GGT upregulation on cancer cell membranes, promoting GGT-mediated cellular uptake of subsequent doses of GLOXmp. In xenograft mouse models, GLOXmp could accumulate in tumors more preferentially due to the Glu_10_ surface decoration. Importantly, repeated administration of GLOXmp reprogrammed tumor cells to upregulate GGT, resulting in enhanced and more effective tumor targeting of sequential doses. At a dose of 20 mg/kg, GLOXmp almost completely eradicated tumors without inducing systemic toxicity, clearly demonstrating the feasibility of self-boosting targeted anticancer therapy via tumor cell self-reprogramming. Given their self-reinforcing receptor modulation, self-boosting targeted delivery, and high therapeutic efficacy, GLOXmp exhibits tremendous potential as a precision anticancer nanomedicine that overcomes tumor heterogeneity.

## Materials and Methods

### Materials

Cinnamaldehyde and N-(2-hydroxyethyl)phthalimide were acquired from Sigma-Aldrich (St. Louis, MO, USA). 2-Hydroxyethyl acrylate, p-toluenesulfonic acid, 4-hydroxybenzyl alcohol, 1,1'-carbonyldiimidazole, benzoyl chloride, PEG-methyl ether acrylate, and 4-dimethylaminopyridine were acquired Alfa-Aesar (Japan). Triethylamine (TEA) was acquired from Junsei (Japan). Tetrahydrofuran (THF), dichloromethane (DCM), benzene, methyl alcohol and dimethyl sulfoixde (DMSO) were acquired from Samchun (Korea).

### Synthesis of polyCA

CA-incorporated diamine** (1)** was synthesized from the reaction of cinnamaldehyde (3.0 g, 22.7 mmol) and N-(2-hydroxyethyl)phthalimide (1.95 g, 10.2 mmol) in the presence of p-toluenesulfonic acid in 250 mL dry benzene during the mechanical stirring. The reaction was performed at 95 °C overnight. The reaction mixture was cooled to room temperature and then given 1.0 mL of TEA to terminate the reaction. The solvent was removed by a rotatory evaporator. Phthalimide-protected CA was obtained from silica gel chromatography with a mixture of ethyl acetate/hexane (1:1).** 1** was prepared by removing the phthalimide protecting groups of phthalimide-protected CA. In brief, phthalimide-protected CA was refluxed in the sodium hydroxide solution at 120 °C overnight. The reaction mixture was washed through funnel extraction using DCM and deionized water. The remaining water was eliminated using anhydrous magnesium sulfate, and the remaining solvent was also eliminated under high vacuum. CA-incorporated diacrylate (**2**) was synthesized from the reaction of cinnamaldehyde (3.0g, 22.7 mmol), 2-hydroxyethyl acrylate (8.25 g, 71.1 mmol), hydroquinone (0.37 g, 3.41 mmol) in the presence of p-toluenesulfonic acid in 250 mL dry benzene during the mechanical stirring. This reaction was performed at 95 °C overnight. To terminate the reaction, 1.0 mL of triethylamine was added. The solvent was eliminated using a rotatory evaporator. **2** was purified by silica gel chromatography using ethyl acetate/hexane (1:4). polyCA was synthesized from the Michael addition reaction of **1**, **2** and PEG-methyl ether acrylate in 100 mL anhydrous DCM at 35 °C for 3 days with mechanical stirring under nitrogen atmosphere. The molar ratio of the reaction was 1.0: 0.9: 0.2. After the Michael addition reaction, the reactants were precipitated in cold hexane. The precipitate was dissolved in anhydrous DCM. In order to prevent crosslinking of polyCA during storage, it was stored in small amount of anhydrous DCM containing a trace of hydroquinone. The chemical structure of **1**, **2** and polyCA was confirmed by ^1^H-NMR (JNM-EX200, JEOL, Japan). The average molecular weight of polyCA was determined through diffusion-ordered NMR spectroscopy using a Bruker AVANCE III HD-400 MHz Fourier transform NMR spectrometer at the Future Energy Convergence Core Center (FECC) in Jeonbuk National University.

### Synthesis of B2C

B2C was synthesized from a three-step reaction, as previously reported. In brief, (hydroxymethyl)phenyl benzoate was synthesized from a reaction of 4-hydroxybenzyl alcohol and benzoyl chloride in DCM containing triethylamine. (Hydroxymethyl)phenyl benzoate reacted with 1,1′-carbonyldiimidazole in DCM to yield 4-(benzoyloxy)benzyl 1H-imidazole-1-carboxoylate. B2C was synthesized from a reaction of (hydroxymethyl)phenyl benzoate and 4-(benzoyloxy)benzyl 1H-imidazole-1-carboxoylate in tetrahydrofuran. The chemical structure of B2C was confirmed by ^1^H-NMR (JNM-EX200, JEOL, Japan).

### Preparation and characterizations of GLOXmp

polyCA (1.0 mg), DSPE-PEG-γGlu_10_ (0.1 mg) and B2C (0.1 mg) were added into 100 μL anhydrous THF. The solution was dropped into 1.0 mL of PBS (pH 7.4) during mechanical stirring. GLOXmp were obtained at a concentration of 1.0 mg/mL after removing THF under vacuum. The size distribution of GLOXmp was determined by dynamic light scattering using a size analyzer (Brookhaven Instrument Corp, Holtsville, NY, USA). The morphology of GLOXmp was observed using a transmission electron microscope (Bio-TEM, Hitachi Corp., Japan) operated at 100KV. For sample preparation, a drop of GLOXmp suspension was placed onto a carbon-coated copper grid and allowed to adsorb for 1 min. Excess liquid was removed with filter paper and the grid was then stained with 2 % (w/v) phosphotungstic acid solution for 30 seconds. After removing the excess stain, the grid was air-dried at room temperature before imaging. The zeta potential of GpolyCA was analyzed by using Zeta-potential Analyzer (ELSZ-2000 series, Otsuka Electronics, Japan). The critical micelle concentration (CMC) of polyCA in pH 7.4 PBS was analyzed by using pyrene as a probe. A microplate reader (Synergy Mx, BioTek Instruments, Winooski, VT, USA) was used to measure their absorbance at 334 nm. The loading content and encapsulation efficiency of GLOXmp were determined by measuring the characteristic absorbance of B2C at 305 nm after the centrifugal filtration (100 kDa).

### Molecular docking

The three-dimensional structure of GGT was obtained from RCSB Protein Data Bank (RCSB PDB). Molecular docking simulation was conducted using the HPEPDOCK 2.0 server (http://huanglab.phys.hust.edu.cn/hpepdock/), a peptide-protein docking platform. The docking simulation was used to predict potential binding interactions and assess the affinity of Glu_10_ for GGT.

### Cell culture

SW620, Huh7, RAW264.7 and TCMK-1 cells were obtained from Korean Cell Line Bank (Korea). Cells were cultured in RPMI medium supplemented with 10% fetal bovine serum and Penicillin-Streptomycin (100 U/mL and 100 μg/mL). Cells were maintained at 37°C in a humidified incubator with 5% CO_2_.

### Cytotoxicity studies

The cytotoxicity of GLOXmp was determined by MTT assay. Cells were seeded in a 12 well plate (2×10^5^ cells/well). Cells with 80% of confluency were treated with B2C, polyCA and GLOXmp for 24 h. Each well was added MTT reagent and incubated for 3 h to form formazan crystal. Formazan crystal was dissolved by DMSO. The cell viability was determined by measuring the absorbance at 570 nm using a microplate reader (Synergy Mx, BioTek Instruments, Winooski, VT, USA).

The effect of combining polyCA micelles and B2C was studied by determining a combination index (CI) calculated from pooled data from three individual experiments each comprising at least three data points for each drug alone and for their combinations. CI for two compounds were be calculated from the following calculation based on Chou and Talalay median effect equation:^37^



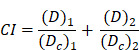



where D_1_ and D_2_ are doses of polyCA micelles and B2C in combination to achieve x% inhibition, whereas D_1x_ and D_2x_ in the denominator represent doses of compounds 1 and 2 to achieve x% inhibition when present alone.

### Determination of intracellular ROS generation

To evaluate the ROS generating ability of polyCA micelles and cinnamaldehyde (CA), SW620 and Huh7 cells were seeded in a glass-bottom dish (SPL Life Sciences, Korea) at a density of 6×10^4^ cells/dish. Cells were treated with polyCA micelles and CA for 12 h. Cells were washed with PBS at twice and then 20 μM DCFH-DA dissolved in DMSO was added to each dish. After 15 min, cells were washed with PBS twice. The fluorescence images of cells were obtained using a confocal laser scanning microscope (Carl Zeiss, Inc., Germany).

### Measurement of the level of intracellular GSH, ATP and nuclear Nrf2

Cells (SW620 and Huh7) were seeded in 6 well plate (5×10^5^ cells/well). Cells with 90% confluency were treated with polyCA micelles, B2C and GLOXmp for 6 h. After washing with PBS, cells were harvested and lysed with 200 μL of cell extraction buffer at 4ºC. Cell lysates were centrifuged at 12,000 rpm for 30 min. The supernatant (10 μL) was taken and mixed with 50 μL of Ellman's reagent (0.5 mM, 5,5′-dithiobis-(2-nitrobenzoic acid)). To determine the level of intracellular GSH, the absorbance at 405 nm was measured using a microplate reader. To measure the level of intracellular ATP, the supernatant (100 μL) was mixed with 100 μL of ATP assay buffer (Biomax, Korea) and incubated for 30 min in dark. The absorbance was measured at 570 nm using a microplate reader. To measure the level of Nrf2, nuclear protein fractions were isolated using the NE-PER™ Nuclear and Cytoplasmic Extraction Reagents (Thermo Scientific) at 4°C. Nrf2 levels in the nuclear extracts were quantified using the human Nrf2 ELISA kit (Invitrogen). Absorbance at 450 nm was measured with a microplate reader, and Nrf2 concentrations were determined by interpolating the absorbance values from a standard curve generated with recombinant Nrf2 protein.

### *In vitro* anticancer activity of GLOXmp

To evaluate apoptotic cell death, SW620 cells were treated with B2C, GpolyCA micelles and GLOXmp. After 24 h of incubation, cells were treated with Annexin V-FITC and propidium iodide purchased from BD Biosciences Pharmigen (San Diego, CA, USA) and incubated for 15 min in dark at room temperature. To examine the effect of GLOXmp on the mitochondrial disruption, JC-1 dye was added to cells treated with B2C, GpolyCA micelles and GLOXmp. Cells were then given 500 μL of 1× binding buffer and the stained cells were transferred to 5 mL of round bottom tubes. The level of apoptotic cell death was quantified using a flow cytometer (FACS Caliber, Becton Dickinson, San Jose, CA).

### Cellular uptake

To verify internalization of GpolyCA micelles, IR780 was used as a fluorescent probe. SW620 cells were seeded in a glass bottom dish (SPL Life Sciences, Korea) at a density of 6×10^4^ cells/dish. IR-780 loaded GpolyCA micelles were added into each confocal dish. At the intended time interval, cells were washed gently with PBS. The fluorescence images of cells were obtained using a confocal laser scanning microscope.

### A mouse xenograft model

SW620 cells (2×10^6^ cells) were subcutaneously injected into the nude BALB/c mice (6 weeks old, Orient Bio, Korea). To examine the biodistribution of GLOXmp, fluorescent GpolyCA micelles were retro-orbitally injected into tumor-bearing mice and the fluorescence images of tumor-bearing mice were obtained using a fluorescence imaging system (Flour i, NeoScience, Korea). When the tumors developed to have a volume 50 mm^3^, the mixture of GpolyCA micelles and B2C (20 mg/kg) or GLOXmp (10 and 20 mg/kg) were administrated injected through retro-orbital injection every 3 days for a total of 10 doses. The body weight and tumor volume of mice were measured every 3 days for 36 days. At the end of the experimentation, tumors and organs were excised for histological examination. Tumor tissues were fixed with formalin and embedded into paraffin. The tissue blocks were sectioned 6 μm thick using a rotatory microtome. Tumor tissues were stained with hematoxylin and eosin (H&E), DHE and terminal deoxynucleotidyl transferase dUTP nick end labeling (TUNEL) and observed under the fluorescence microscope (Eclipse, Nikon, Japan).

To evaluate the self-boosting tumor targeting of GLOXmp, tumor-bearing mice were injected with GLOXmp (5 mg/kg) 2 or 5 times. One day after the last dosing, fluorescent GpolyCA micelles were intravenously injected and fluorescence images were obtained. Tumors were also excised to determine the intratumoral level of GGT. The tissue sections were stained with fluorescent anti GGT-antibody and observed under the fluorescence microscope. The studies were approved by the Institutional Animal Care and Use Committee (JBNU 2022-069) and conducted under the guidance of Laboratory Animal Research Center of Jeonbuk National University.

### Biosafety of GLOXmp

Healthy Balb/c mice were retro-orbitally injected with 20 mg/kg of GLOXmp every two days for 14 days. Blood and major organs were collected on day 16. The blood levels of ALT, AST, creatinine and BUN were determined using ALT and AST kit (Asan Pharma, Korea), creatinine assay kit (Abcam, Cambridge, UK), and BUN assay kit (Abbexa Ltd, Sugar Land, TX, US). Major organs were excised and tissue sections were stained with H&E for histological examination.

### Statistical analyses

Values were expressed as mean ± SD (standard deviation). Comparison between groups was conducted with one-way ANOVA using GraphPad Prism 5.0 (San Diego, CA, USA). Probability (*p*) value of < 0.05 was statistically considered significant.

## Supplementary Material

Supplementary figures.

## Abbreviations

GGT: gamma-glutamyl transferase
ROS: reactive oxygen species
Glu: glutamic acid
GSH: glutathione
CA: cinnamaldehyde
B2C: benzoyloxy dibenzyl carbonate
GLOXmp: glutamic acid-coated oxidative stress nanoamplifier
DSPE: 1,2-distearoyl-sn-glycero-3-phosphoethanolamine
PEG: polyethylene glycol
DSPE-PEG-Glu_10_: 1,2-distearoyl-sn-glycero-3-phosphoethanolamine)-Polyethylene glycol-Glu_10_
QM: quinone methide
CMC: critical micelle concentration
TEM: transmission electron microscopy
DCFH-DA: dichlorodihydrofluorescein-diacetate
NAC: N-acetyl cysteine
CI: combination index
MTT: 3-(4,5-dimethylthiazol-2-yl)-2,5-diphenyltetrazolium bromide
PI: propidium iodide
FITC: fluorescein isothiocyanate
Nrf2: nuclear factor E2-related factor 2
DHE: dihydroethidium
TUNEL: terminal deoxynucleotidyl transferase dUTP nick end labeling
PGA: polyglutamic acid
ALT: alanine aminotransferase
AST: aspartate aminotransferase
BUN: blood urine nitrogen
ELISA: enzyme-linked immunosorbent assay**.**
