# Supplementary material for: Self-boosting targeted anticancer therapy via cancer cell self-reprogramming with GGT-targeting oxidative stress nanoamplifiers
Source: Theranostics. 2026 Jan 1;16(4):1925–40. doi: 10.7150/thno.120954 (PMC12680591; doi:10.7150/thno.120954)
Supplement: Supplementary file 1 — Supplementary figures. [file thnov16p1925s1.pdf]

Self-boosting targeted anticancer therapy *via* cancer cell self-reprogramming with GGT-targeting oxidative stress nanoamplifiers

*Sujin Kim[1], Suyeon Lee[1], Manseok Yang[1], Seungwon Jung[1], Nanhee Song[1], Nuri Kim[1], Hanui Jo[1], Seunga Lee[1], Chaihong Nah[2], Seong-Cheol Park[3], Dongwon Lee[1,4,]**

[1] Department of Bionanotechnology and Bioconvergence Engineering, Jeonbuk National University, Jeonju, Jeonbuk 54896, Republic of Korea
[2] Department of Chemistry, Lehigh University, Bethlehem, PA 18015, USA
[3] Department of Polymer Engineering, Sunchon National University, Sunchon, Jeonnam 57922, Republic of Korea
[4] Department of Polymer·Nano Science and Technology, Jeonbuk National University, Jeonju, Jeonbuk 54896, Republic of Korea
* Corresponding author: Dongwon Lee, E-mail: dlee@jbnu.ac.kr

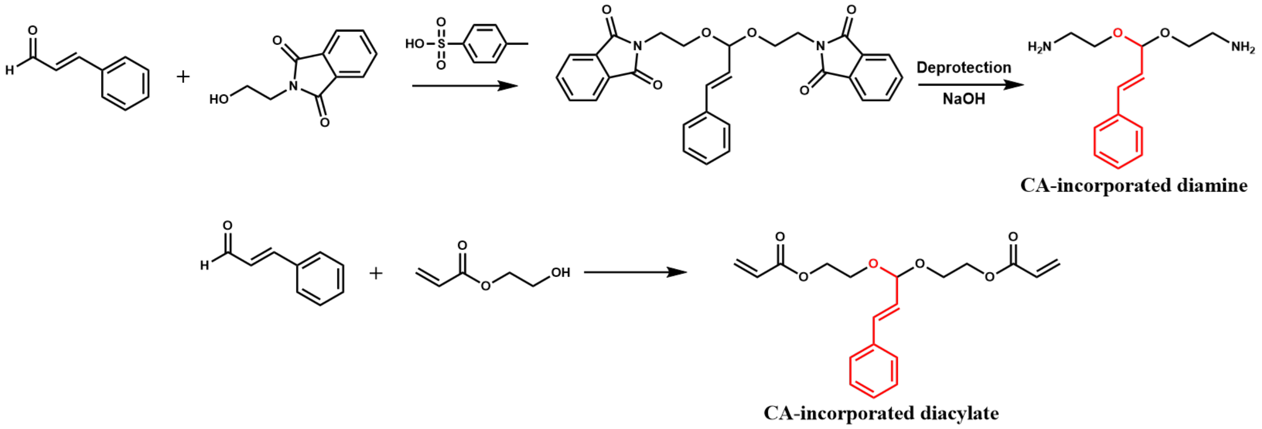

Figure S1. Synthesis of CA-incorporated diamine and CA-incorporated diacrylate.

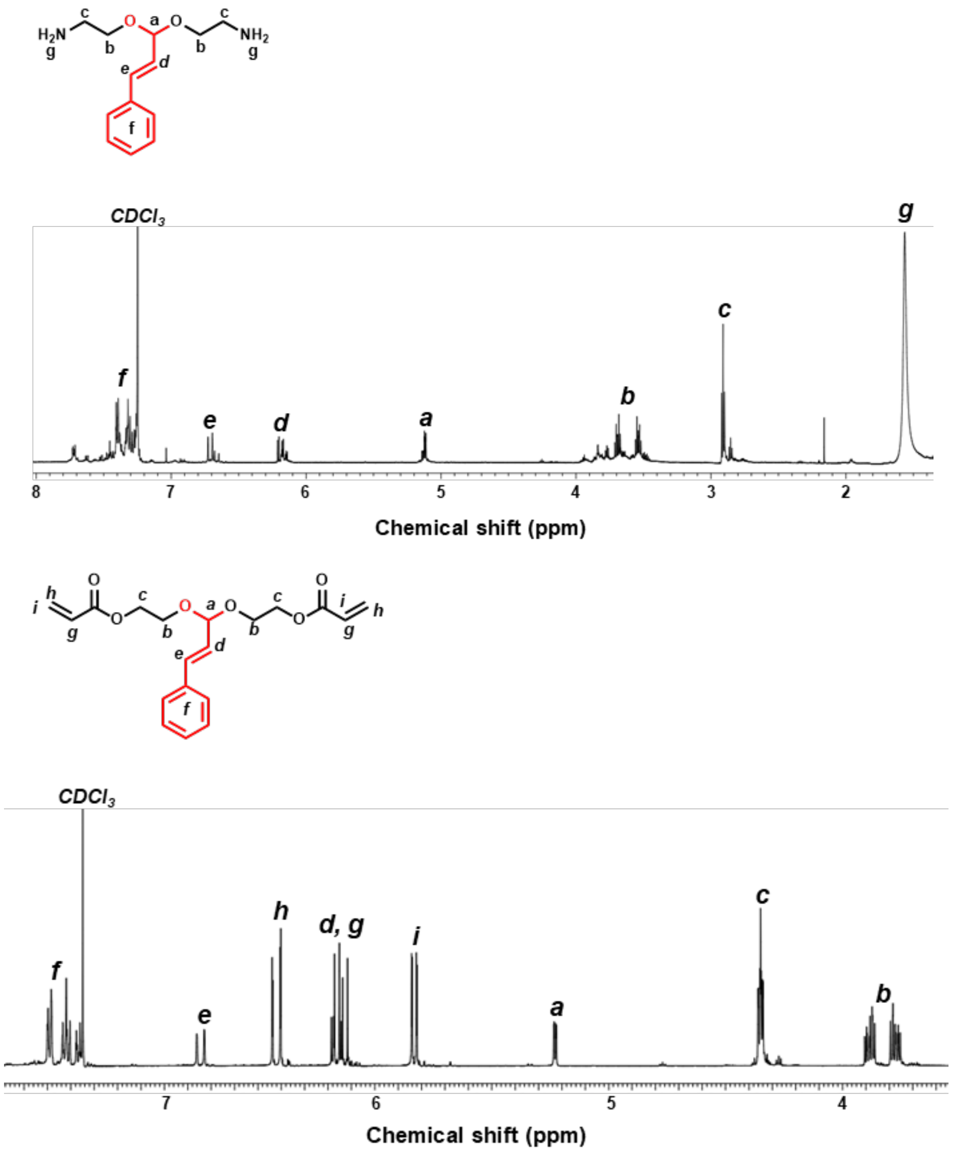

Figure S2. NMR spectra of CA-incorporated diamine and CA-incorporated diacrylate.

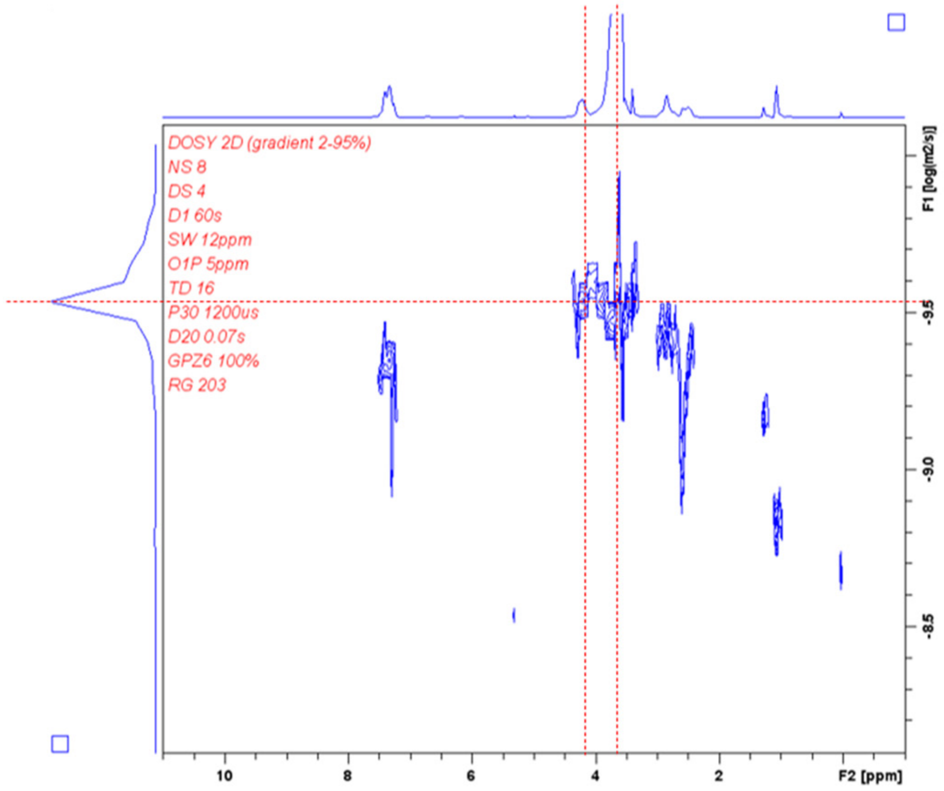

Figure S3. DOSY-NMR of polyCA.

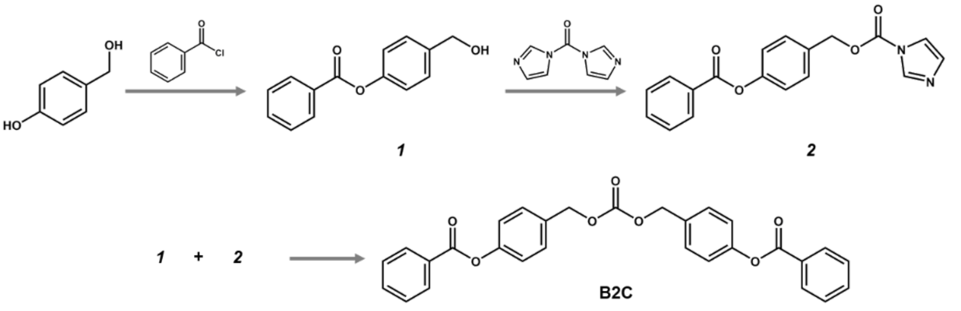

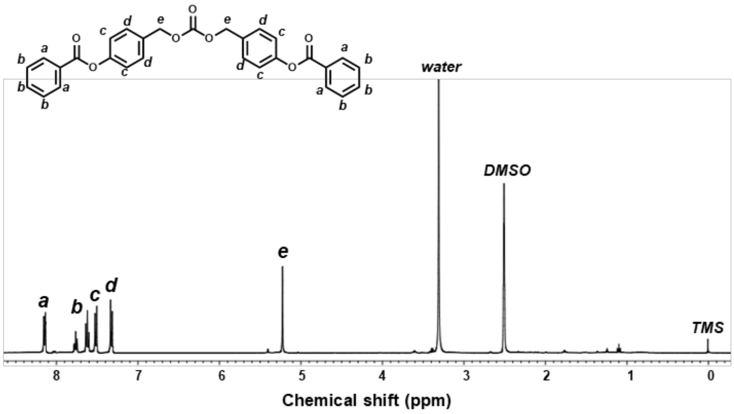

Figure S4. NMR spectrum of B2C.

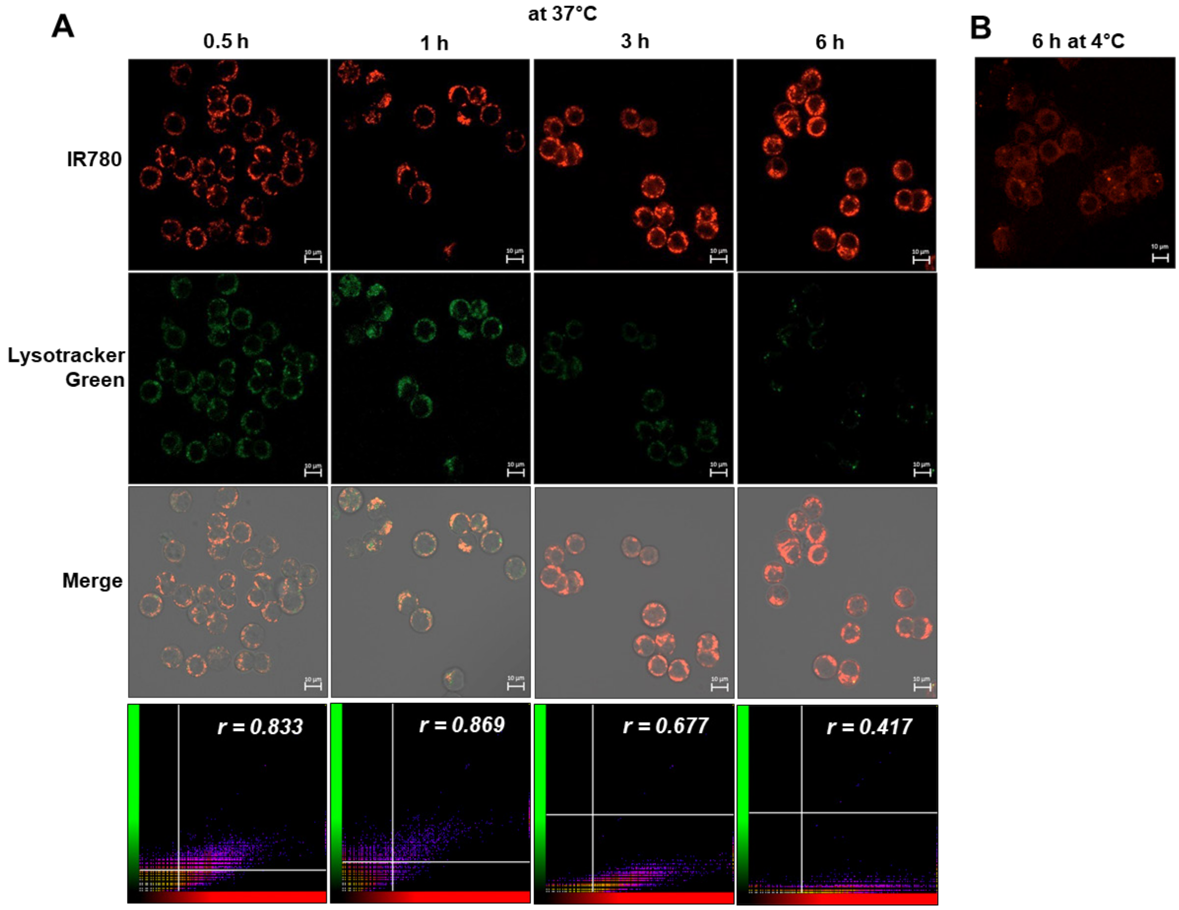

Figure S5. Cellular uptake of IR780-loaded GpolyCA micelles. (A) Fluorescence images of SW620 cells incubated with GpolyCA micelles for varying durations. Acidic compartments such as endosomes were stained with Lysotracker Green. The bottom panels show the Pearson correlation coefficient of co-localization of green and red signals. (B) Fluorescence images of SW620 cells incubated with GpolyCA micelles for 6 h at 4 °C.

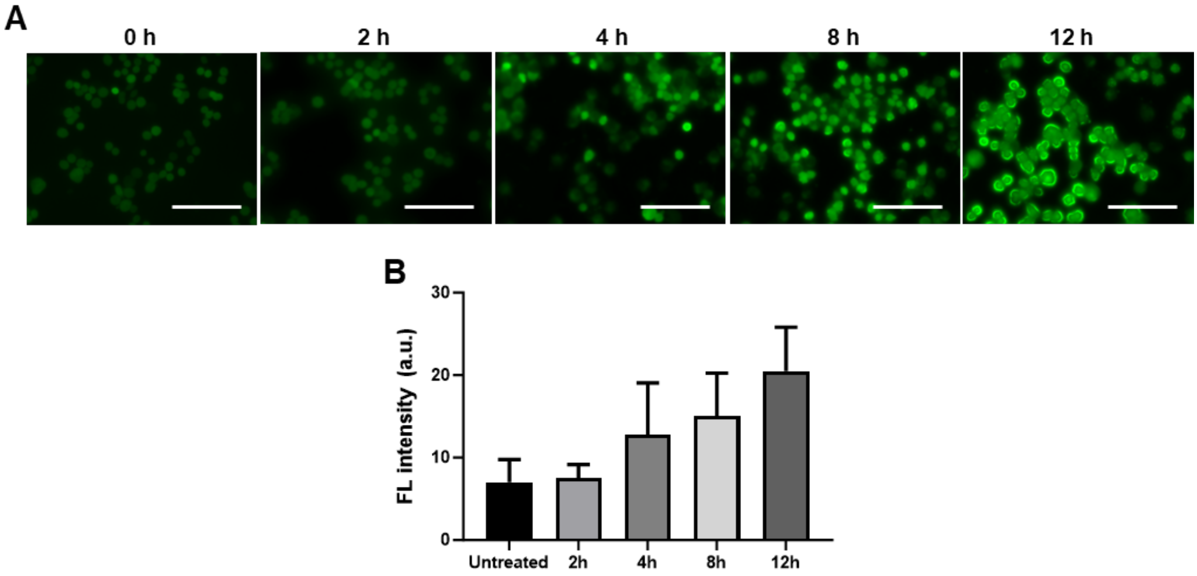

Figure S6. ROS generation by polyCA micelles in SW620 cells. (A) Fluorescence images of cells generating ROS at different time points. (B) Quantitative ROS generation rate in cells. Values are mean ± s.d. (n = 3)

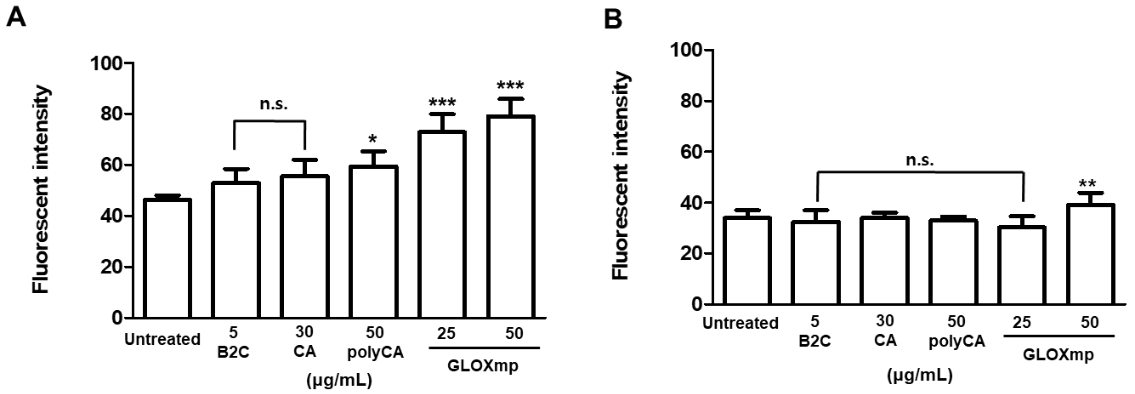

Figure S7. Quantification of the level of ROS in (A) Huh7 cells and (B) TCMK-1 cells treated with CA, polyCA micelles and GLOXmp. Values are mean ± s.d. (n = 4). *** $p < 0.001$, ** $p < 0.01$, * $p < 0.05$ relative to Untreated group.

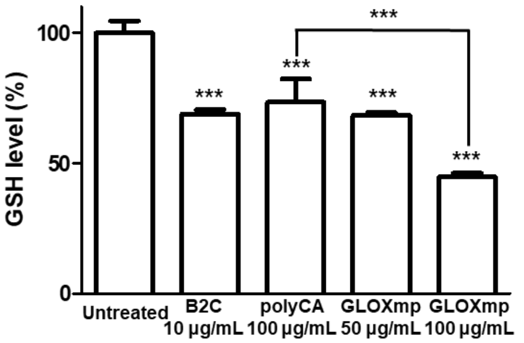

Figure S8. The intracellular level of GSH in Huh7 cells treated with CA, polyCA and GLOXmp. Values are mean ± s.d. (n = 4). *** $p < 0.001$, ** $p < 0.01$ relative to Untreated group.

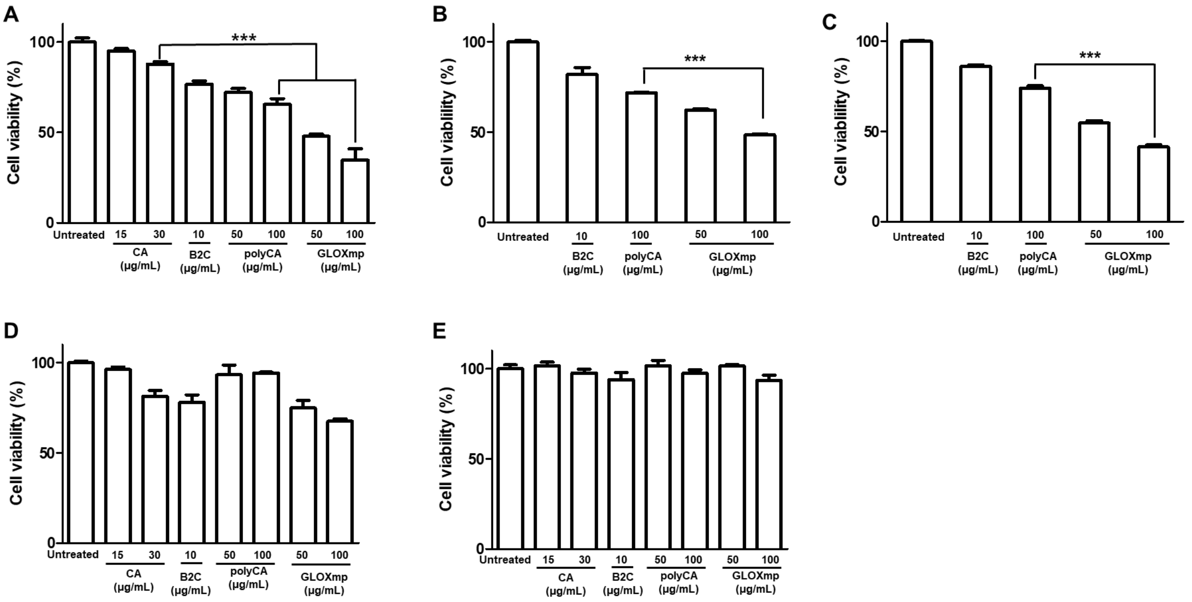

Figure S9. Cytotoxicity of GLOXmp against various cells. (A) Huh7 cells, (B) MCF-7 cells, (C) A549 cells, (D) RAW264.7 cells, (E) TCMK-1 cells. Values are mean ± s.d. (n = 4). *** p < 0.001.

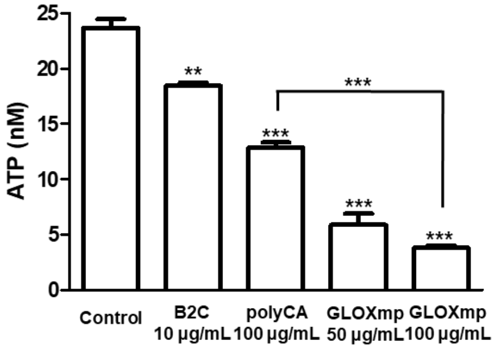

Figure S10. The intracellular level of ATP in Huh7 cells after treatment with polyCA, B2C, GLOXmp. Values are mean ± s.d. (n = 4). *** $p < 0.001$, ** $p < 0.01$ relative to Control group.

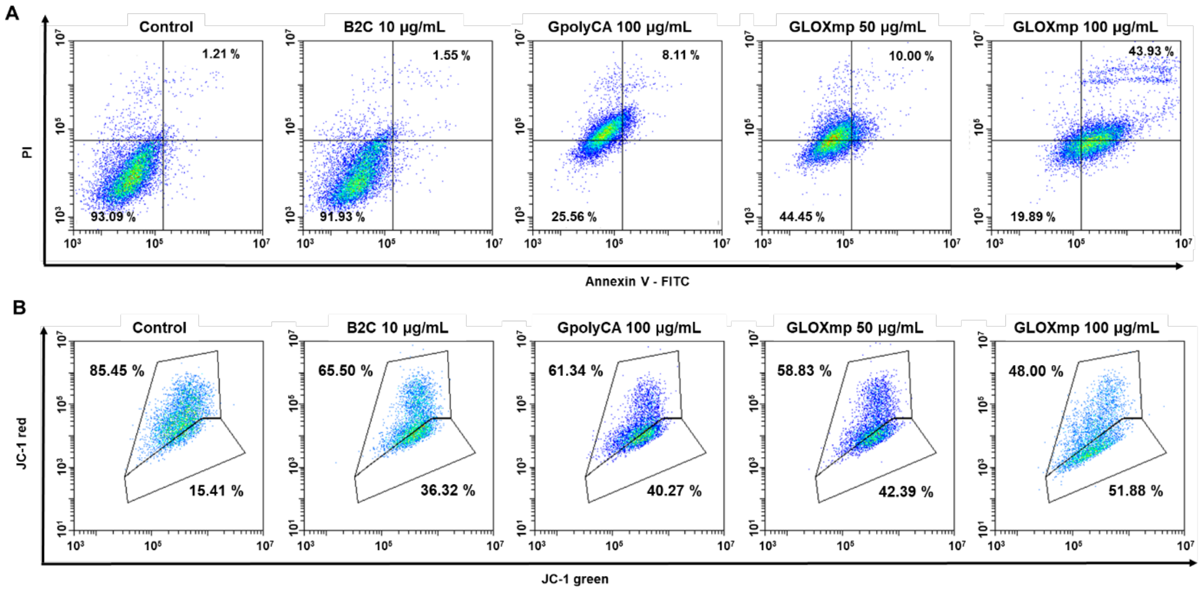

Figure S11. Flow cytometric analysis of Huh7 cells stained with (A) FITC-Annexin V and (B) JC-1 dye and after the treatment with GpolyCA and B2C, GLOXmp.

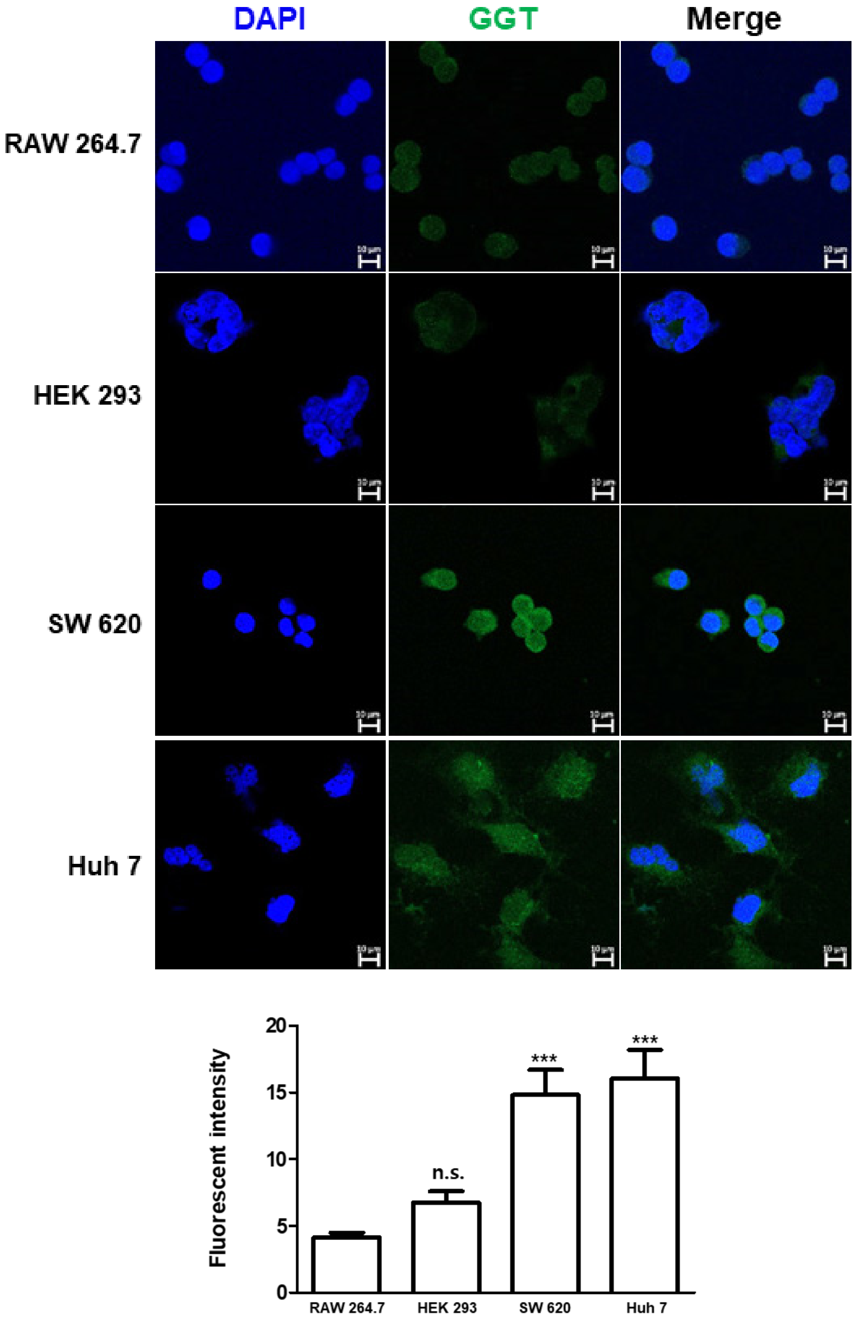

Figure S12. Fluorescence images of various cells stained with anti GGT-antibody and quantification of the level of GGT. Values are mean ± s.d. (n = 4). *** $p < 0.001$, relative to RAW264.7 cell.

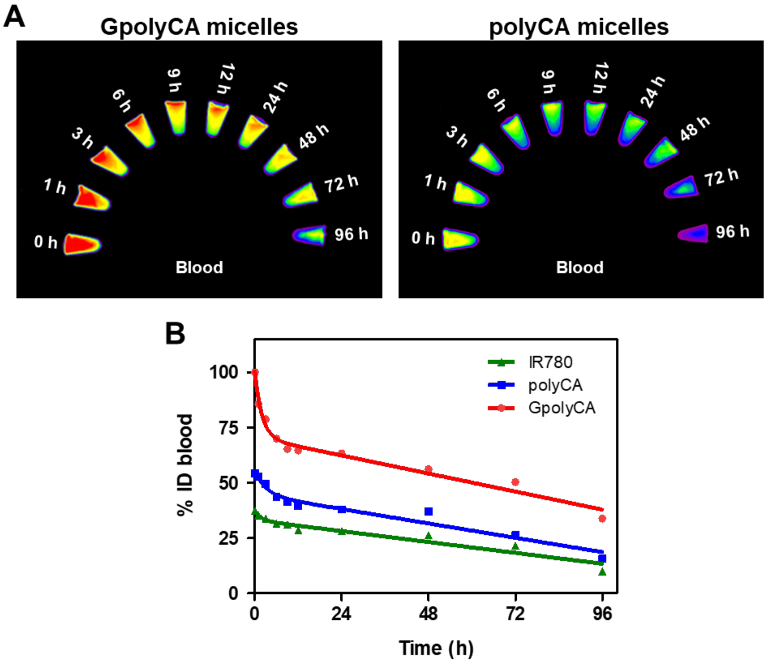

Figure S13. Pharmacokinetics of GpolyCA micelles and polyCA micelles that encapsulate IR780. (A) Fluorescence images of blood taken from mice at intended time points. (B) Quantified fluorescence intensity in blood as a function of time.

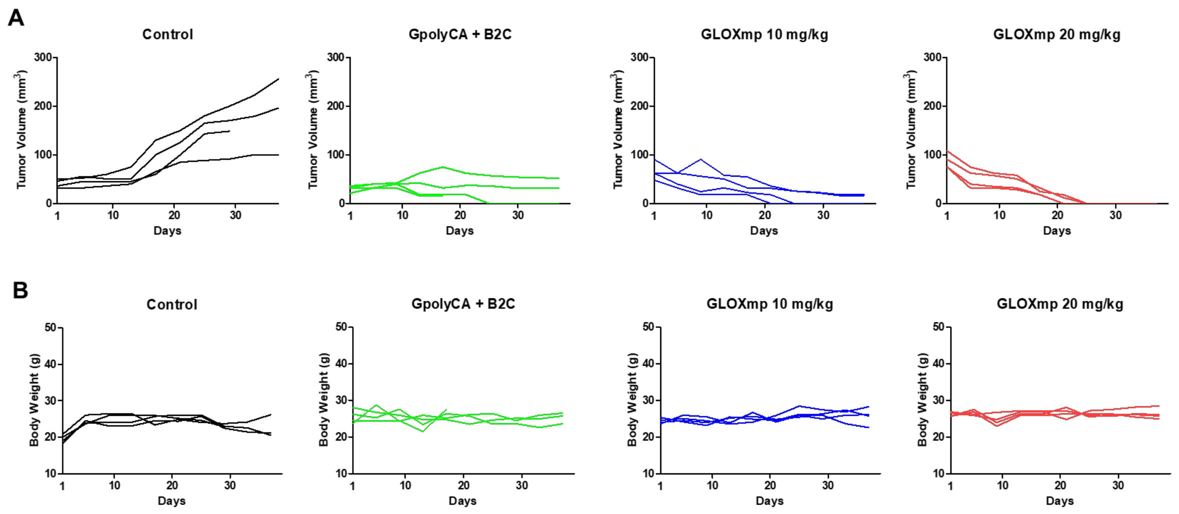

Figure S14. Changes of (A) tumor volumes and (B) body weight during the treatment.

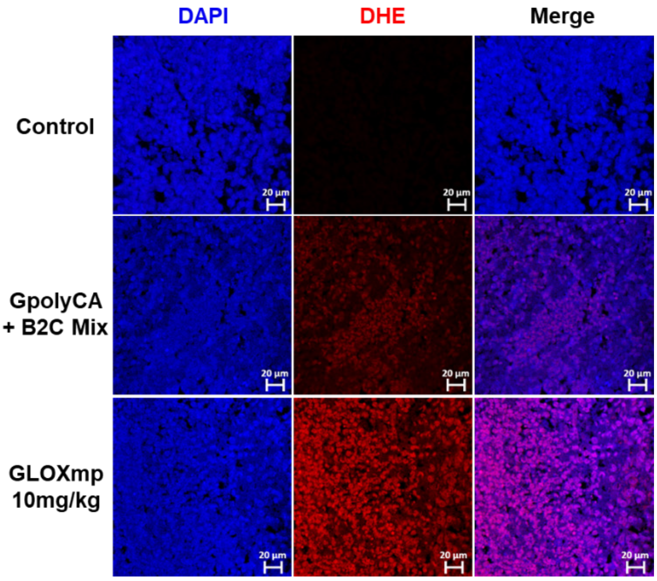

Figure S15. Tumor tissues stained with DHE.

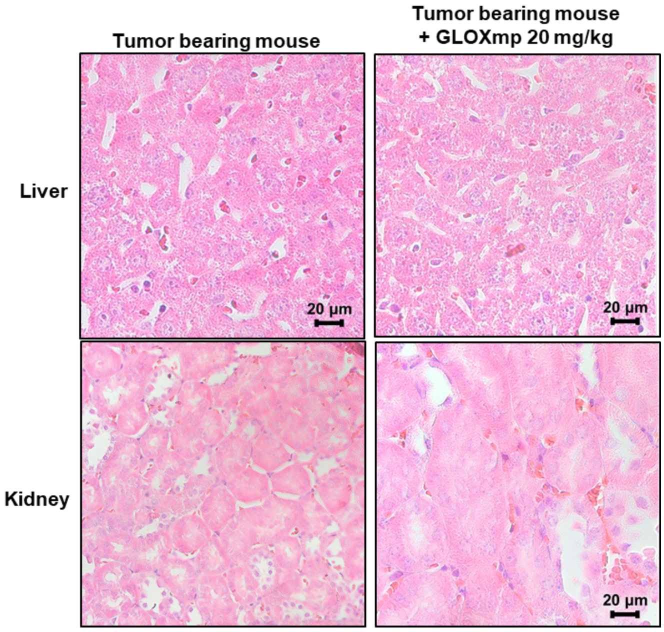

Figure S16. Histological examination of liver and kidney stained with H&E.

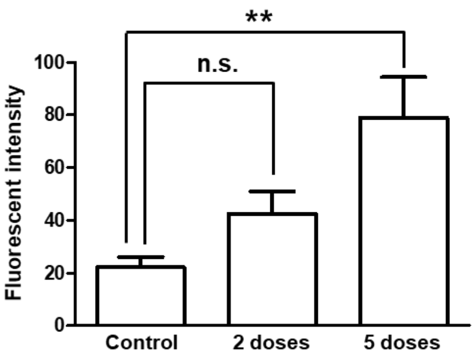

Figure S17. The level of GGT expression in tumor tissues. Values are mean ± s.d. ($n = 4$). **$p < 0.01$. n.s. denotes non-significant.

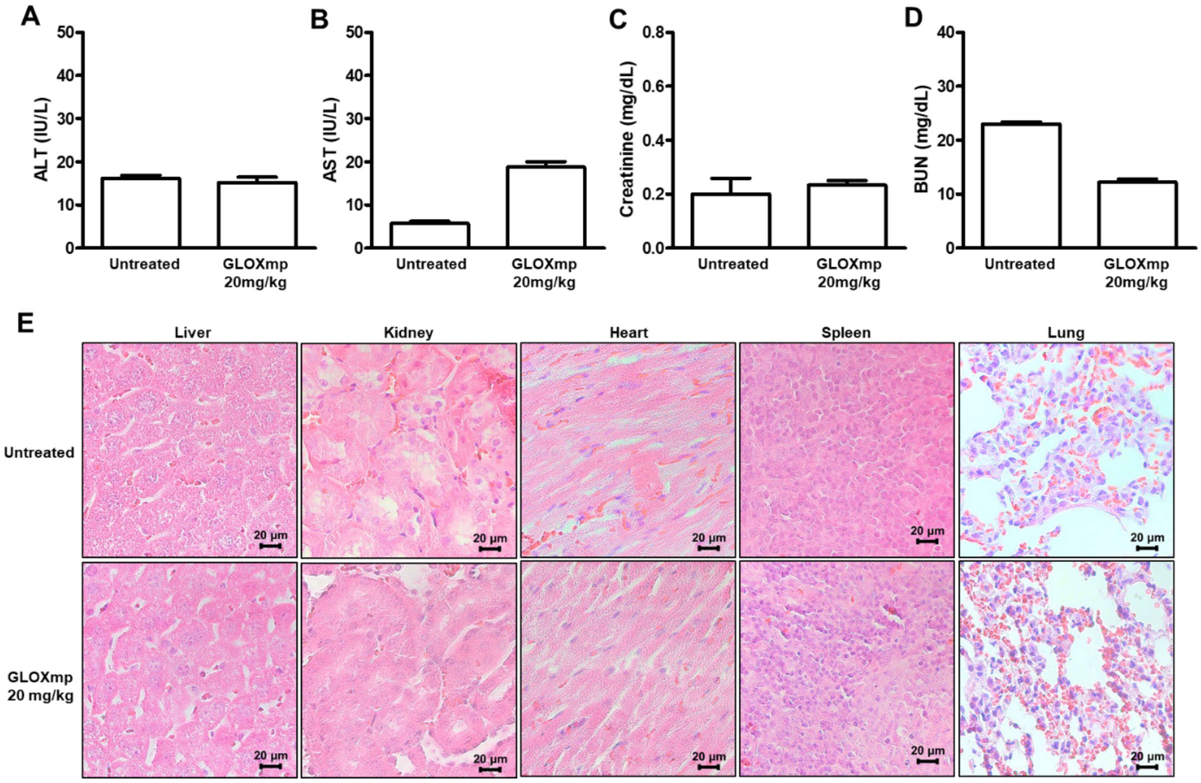

Figure S18. Biosafety assay of GLOXmp. The blood level of (A) ALT, (B) AST, (C) Creatinine, and (D) BUN. Values are mean ± s.d. (n = 4). (E) Micrographs of major tissue sections stained with H&E.
